# The PDZ domain of EpsC is required for extracellular secretion of VesB by the Type II secretion system in *Vibrio cholerae*

**DOI:** 10.1128/jb.00144-25

**Published:** 2025-07-14

**Authors:** Austin Shannon, Tanya Johnson, Cameron S. Roberts, Catherine T. Chaton, Konstantin V. Korotkov, Maria Sandkvist

**Affiliations:** 1 Department of Microbiology and Immunology, University of Michigan 242912, Ann Arbor, Michigan, USA; 2 Department of Molecular and Cellular Biochemistry, University of Kentucky 167091, Lexington, Kentucky, USA; National Institutes of Health, Bethesda, Maryland, USA

**Keywords:** *Vibrio cholerae*, type II secretion system, T2SS, GspC, PDZ domain, secretion signal, Ig domain, serine protease, substrate selection, periplasmic retention, AviTag biotinylation

## Abstract

The Type II Secretion System (T2SS) of the Gram-negative pathogen *Vibrio cholerae* is considered a virulence factor primarily because it facilitates the secretion of cholera toxin upon arrival of this pathogen in the human intestinal lumen, resulting in the profuse watery diarrhea associated with infection. Results from transposon mutagenesis screens suggest that the *V. cholerae eps* genes that code for the T2SS are essential. The deletion of these genes impairs the secretion of the T2SS’s nearly two dozen protein substrates, leading to the selection of suppressor mutations, extracytoplasmic stress, and poor growth of the mutant bacteria. Here, we explore the role of the C-terminal PDZ domain of the T2SS component EpsC, and why transposon insertions in *pdz* do not affect bacterial viability unlike insertions in most other *eps* genes. Our data show that the deletion of *pdz* does not affect the growth of *V. cholerae,* and we demonstrate via mass spectrometry that the serine protease VesB is the only known T2SS substrate fully reliant on PDZ for secretion. We confirm this finding with immuno- and enzyme assays and show that the immunoglobulin (Ig)-like fold of VesB contains PDZ-dependent secretion information that can be grafted onto β-lactamase, causing this normally periplasmic protein to be secreted in a PDZ-dependent manner. This work adds to a growing body of evidence about substrate selection by the T2SS and reinforces the view that the C-terminal domain of EpsC and its homologs are required for the secretion of only a subset of T2SS substrates within a given species.

**IMPORTANCE:**

The T2SS is common in Gram-negative pathogens, facilitating the secretion of various toxins and enzymes; however, the mechanisms of substrate selection and secretion remain poorly understood. Here, we demonstrate that the C-terminal PDZ domain of the T2SS “clamp” protein EpsC is only required for secretion of VesB in *V. cholerae* and that VesB’s Ig-fold contains PDZ-dependent secretion information that can be functionally grafted onto a non-secreted periplasmic protein.

## INTRODUCTION

The Type II Secretion System (T2SS) is a multi-protein nanomachine that secretes hydrolytic enzymes and virulence factors from diverse Gram-negative bacterial species inhabiting very different environmental niches. Importantly, T2SSs are utilized by plant and human pathogens to facilitate host immune evasion, biofilm formation, competition with other species, and to extract resources from their hosts (1, 2). The enteric human pathogen *Vibrio cholerae* utilizes its T2SS in the aquatic environment to secrete chitin-binding protein GbpA and chitinases to extract nutrients from arthropods (3–5). When *V. cholerae* enters the human gut through the consumption of contaminated food or water, the T2SS is essential to its pathogenesis because the T2SS facilitates secretion of its primary virulence factor, cholera toxin (CT), as well as proteases and numerous other enzymes that help the bacteria avoid the innate immune response and scavenge resources (4–9).

Secretion through the T2SS requires that precursor substrates first enter the periplasm through the Sec or Tat transporters and then fold into their native conformation following removal of their signal peptides (10–12). Substrates are then recognized by the T2SS machinery and delivered to the extracellular space as fully soluble or surface-localized proteins (4, 13–15). The 15 components of the T2SS in *V. cholerae* are each designated as “extracellular protein secretion X” (Eps_), and they together create an ATPase-powered endopilus complex spanning the inner membrane (IM) that is coupled to an outer membrane (OM) pore called a secretin (16–19).

EpsC, an essential T2SS component, is sometimes referred to as the “clamp” protein because it holds the larger IM and OM structures together (20–22). EpsC has three distinct domains: an N-terminal transmembrane (TM) helix that associates with the IM endopilus platform, followed by a periplasmic homology region (HR domain) that binds the N0 domain of the T2SS secretin pore (EpsD), and a C-terminal PDZ domain of unknown function (21). Mutations of key residues involved in EpsC/EpsD interaction halt protein secretion and disperse puncta formed by GFP-conjugated EpsC as observed via fluorescence microscopy (22). It is understood that the transmembrane and HR domains of EpsC have a global impact on type II secretion because these regions facilitate the connection between EpsD and the T2SS endopilus platform in the inner membrane. While the role of EpsC’s PDZ domain is not well understood, PDZ domains are considered protein-protein interaction domains and are ubiquitous in nature (23). Named for the first three proteins identified with this domain, PDZ domains are commonly found in membrane-associated complexes in eukaryotes, facilitating close interactions with partner proteins canonically through binding at the extreme C-terminus (24, 25). In bacteria, PDZ domains are commonly part of proteases, such as DegS (a bacterial homolog to HtrA), which has a PDZ domain that senses misfolded proteins, leading to the activation of the protease domain and cleavage of anti-sigma factor RseA in *Escherichia coli* (26–28). PDZ’s association with protein binding and signal transduction has led to speculation that this domain in the T2SS may be involved in substrate selection (29).

The genes for the T2SS system in *V. cholerae* have been designated as essential in saturation transposon mutagenesis screens (30, 31). This finding has been corroborated by experimental evidence from our lab and others, demonstrating that deleting the entire *eps* operon or individual genes of the system (*epsC, epsD, epsE*, etc.) in *V. cholerae* is challenging and selects for secondary mutations that may suppress the lethal phenotype (32). These deletions not only prevent substrate secretion but also cause severe growth defects and cell integrity alterations that result in an extracellular increase of cytosolic and periplasmic proteins (33, 34). Interestingly, *epsC* was identified as “domain essential” by Chao et al. (30), meaning that transposon insertions were only recovered in one region of the gene (~300 nucleotides) encoding the C-terminal PDZ domain. Mutants with transposons in *epsC* regions that code for the TM or the HR domains upstream of PDZ were not obtained (30). The C-terminal PDZ region of EpsC is particularly interesting because it is absent in the EpsC homolog PilP of the structurally related type-IV pilus (T4P), suggesting a specific role in secretion (35). This region exhibits significant variability among the T2SSs of different species. For instance, while many T2SSs have a PDZ domain like *V. cholerae*, LspC of *Legionella pneumophila* lacks this domain, and XcpP of *Pseudomonas aeruginosa* has a coiled-coil domain at this position (36–38). The PDZ domain of the EpsC homolog OutC, in the plant pathogen *Dickeya dadantii,* was found to be required for the secretion of some but not all T2SS substrates, but it is currently unknown whether this holds true for other species (39–41).

Here, we demonstrate that while deleting portions of the essential T2SS component EpsC leads to the aforementioned problems with growth, the deletion of EpsC’s C-terminal PDZ domain does not. Furthermore, through quantitative mass spectrometry, immunoassays, and enzyme assays, we demonstrate that the PDZ domain deletion significantly reduces the secretion of serine protease VesB, while marginally affecting the secretion of the VesB homologs, VesA and VesC. We also show that the VesB Ig-fold domain can be grafted onto the periplasmic β-lactamase, promoting its extracellular secretion in a PDZ-dependent manner.

## RESULTS

### Deletion of the C-terminal PDZ domain of EpsC impedes serine protease secretion but does not affect growth

**Fig 1:**
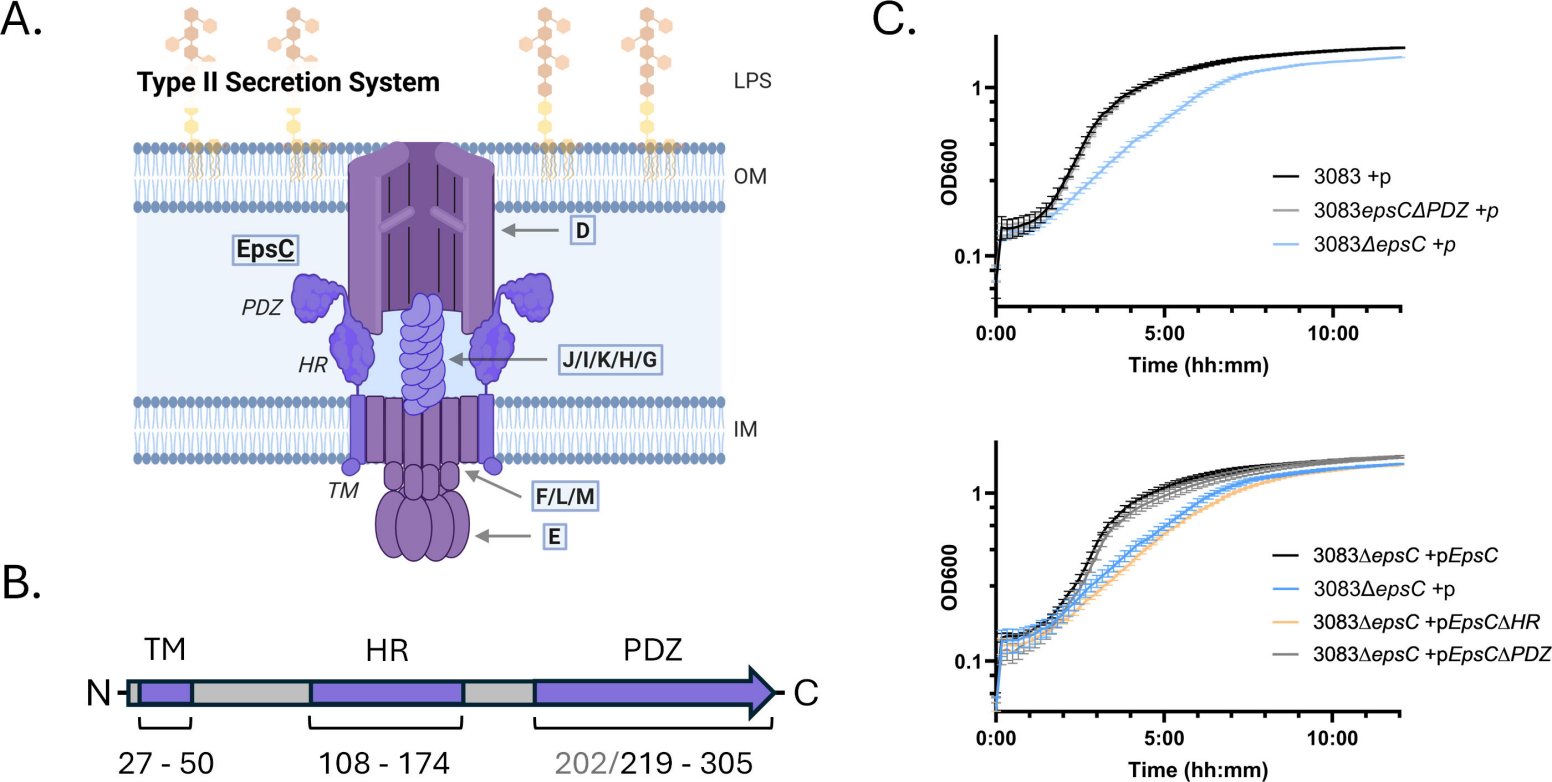
Deletion of the TM or HR region of the *epsC* gene causes a growth defect, while deletion of PDZ does not affect growth. (A) Schematic model of the Type II Secretion System (T2SS) with each protein component labeled with its associated letter designation (e.g., EpsD is labeled “D”). “OM” and “IM” are outer- and inner membranes, respectively. The structured domains of protein component EpsC are also indicated, but EpsA, EpsB, EpsN, and the pre-pilin peptidase EpsO are not shown. This diagram was created using BioRender. (B) Organization of structured domains in EpsC. “TM” indicates the transmembrane domain, “HR” is the homology region, and “PDZ” is the PDZ domain. While the organized structural boundaries of the EpsC PDZ domain begins at amino acid 202 (“long” PDZ), a crystal structure has also been determined of PDZ that lacks the α1 helix and starting instead at 219 (“short” PDZ). (C) Overnight growth of WT *V. cholerae* (strain 3083) with genomic *epsC* modifications and empty vector (P) or ectopic expression of different EpsC constructs in an isogenic *epsC::kan* mutant. All strains were grown in LB at 37°C with agitation and monitored by OD_600_ over time. Data represent the mean +/− the standard deviation of three biological replicates each assayed in triplicate.

Previously published transposon mutagenesis screening suggested that disrupting the PDZ domain of EpsC does not affect the growth of *V. cholerae*, unlike disruption of other T2SS components or even disruption of other regions of EpsC (Fig. 1A and B) (30, 31). To better understand why the T2SS PDZ domain appears to be dispensable in *V. cholerae*, we first deleted the *pdz* region (coding for amino acids Q203-F305) from the genome or disrupted the entire *epsC* gene through replacement with a kanamycin cassette (*epsC::kan*) and analyzed complementation of growth with different *epsC* plasmid constructs. Consistent with transposon screening and previous work on *V. cholerae* T2SS mutants, 3083∆*epsC* exhibited a growth defect in LB at 37°C. In contrast, 3083*epsC∆PDZ* growth was comparable to that of wild-type (WT) *V. cholerae* 3083 (Fig. 1C). The same phenotype was observed in a 3083∆*epsC* background where an *epsC* construct with a disrupted HR-coding region was unable to restore WT-level growth in LB, while expression of full-length *epsC* or *epsC*∆*pdz* resulted in complementation of the growth defect (Fig. 1C). These data indicate that, unlike other T2SS mutations, deletion of the PDZ domain is well-tolerated in *V. cholerae*.

**Fig 2:**
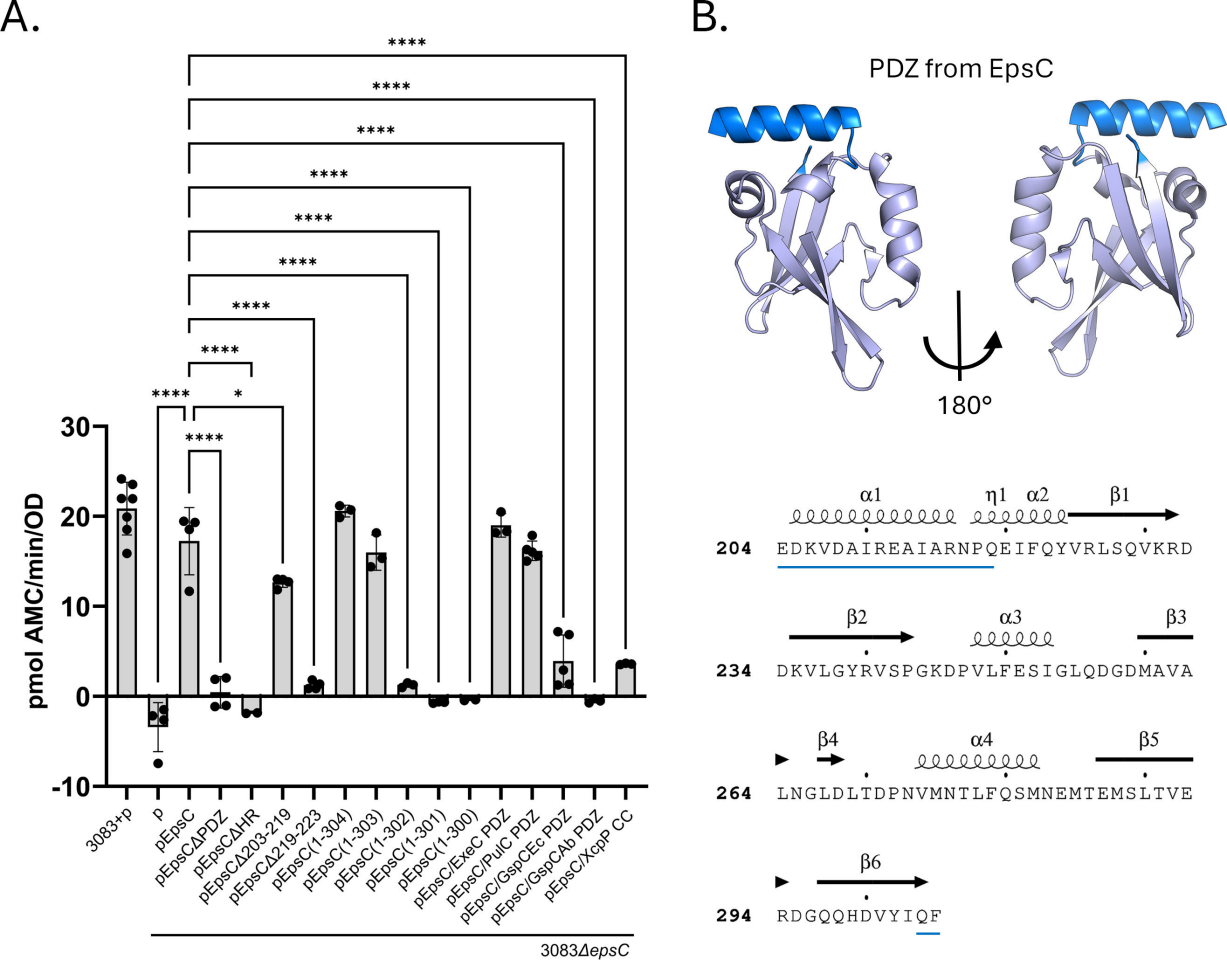
Complementation of an ∆*epsC* mutant with truncated EpsC and PDZ chimera. (**A**) Culture supernatants from overnight cultures of WT and *∆epsC* mutant strains of *V. cholerae* 3083 containing empty vector (P) or plasmids coding for different EpsC variants were analyzed for serine protease activity using a methylcoumarin-conjugated peptide (Boc-Gln-Ala-Arg-AMC). “p” represents empty vector, and the various “pEpsC/__” plasmids express EpsC chimeras with PDZs from EpsC homologues in *A. hydrophila* (ExeC), *K. pneumoniae* (PulC), *E. coli* (GspCEc), *A. baumannii* (GspCAb), and the coiled-coil of P. aeruginosa (XcpP)—see Fig. S1 for additional information. Mean +/− standard deviation of biological replicates each with technical triplicates are represented (*n* ≥ 3). All data were analyzed by an ordinary one-way ANOVA with a Dunnet test comparing each sample to 3083*∆epsC* + pEpsC. While all data were included in the ANOVA analysis, only significant differences are indicated (*P ≤ 0.05, ****P ≤ 0.0001). (**B**) Crystal structure (PDB: 2I4S) of the long PDZ from *V. cholerae* EpsC is on top with the associated amino acid sequence underneath indicating secondary structures (ESPript 3.0). VesB is still secreted when the regions highlighted in blue and underlined in the sequence are deleted.

Our lab has previously shown that measuring serine protease activity in culture supernatants using a fluorescent methylcoumarin-conjugated peptide (Boc-Gln-Ala-Arg-AMC) as a substrate is an appropriate way to monitor protein secretion in *V. cholerae* since the T2SS is required for the extracellular transport of three serine proteases (42). We used this assay to measure the impact of various *epsC* modifications on secretion, confirming that the *epsC::kan* mutant exhibiting poor growth is also nonfunctional for serine protease secretion (Fig. 2A). The same is true for the ectopic expression of *epsC∆hr* in the *epsC::kan* mutant. Interestingly, we found that even with a growth rate comparable to WT *V. cholerae*, the *epsC∆pdz* mutant is unable to secrete active serine protease. Recognizing the dramatic effect of PDZ domain deletion on protease activity, we sought to further define the functional boundaries of the PDZ domain. Previous work on the PDZ domain of EpsC described the crystal structures of two different PDZ constructs: a “long” PDZ (G204-F305—Protein Data Bank identifier [PDB] 2I4S) beginning after the unstructured linker between HR and PDZ, and a truncated “short” PDZ (Q219-F305—PDB: 2I6V) that excludes the α1 helix (Fig. 2B) (21). Our data show that the α1 helix of PDZ is not required for serine protease secretion. In contrast, removal of the α2 helix and C-terminal truncations beyond the last two amino acids (Q304 and F305) do interfere with secretion (Fig. 2A). Additionally, we created EpsC chimera by replacing the EpsC PDZ domain with PDZ from EpsC homologs ExeC in *Aeromonas hydrophila* (43% sequence identity with the EpsC PDZ domain via structure-based alignment)*,* PulC in *Klebsiella pneumoniae* (47% identity)*,* GspC_Ec_ in *Escherichia coli* (19% identity)*,* and GspC_Ab_ in *Acinetobacter baumannii* (20% identity) *or with* the coiled-coil domain of the EpsC homologue XcpP from *P. aeruginosa* (Fig. S1). Both the PDZ from *A. hydrophila* and *K. pneumoniae* complemented the *epsC::kan* mutant, whereas the other three chimeras were unable to restore serine protease secretion (Fig. 2A). All PDZ modifications tested (chimeras and truncations) complemented the growth phenotype of the *epsC::kan* mutant, suggesting that EpsC constructs with these PDZ modifications do not negatively impact the function of the TM or HR domains (Fig. S2). Taken together, these data more precisely define the PDZ domain required for secretion of serine proteases in *V. cholerae*. They also suggest that species-specific interactions of the PDZ domain are necessary for productive secretion in *V. cholerae* and raise the possibility that the PDZ domains of *A. hydrophila* and *K. pneumoniae* are involved in the secretion of proteins with similar properties to *V. cholerae* serine proteases.

### Mass spectrometry reveals that VesB is the only T2SS substrate that requires PDZ for secretion

**Fig 3:**
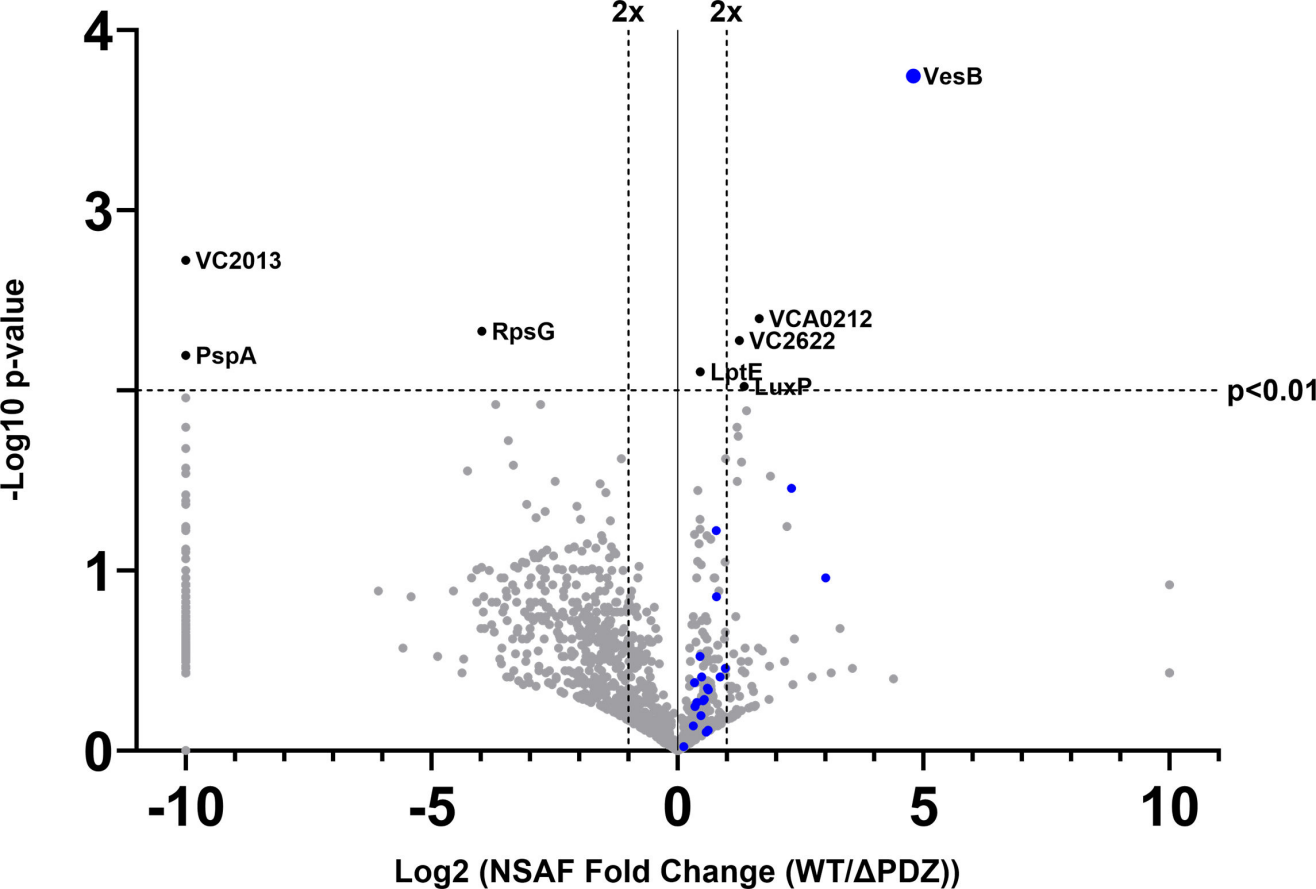
Mass spectrometry analysis of PDZ mutant shows minimal differences compared to WT *V. cholerae*. Volcano plot displaying LC/MS-MS data of proteins detected in the supernatants of WT and *epsC∆PDZ V. cholerae* N16961 strains (*n* = 3). Fold change of the Normalized Spectral Abundance Factor (NSAF) is shown on the *x*-axis and significance as determined by a Student’s *t*-test is on the *y*-axis. −10 fold-change was manually assigned to proteins that were only detected in the culture supernatant of the PDZ mutant, and 10 is assigned to those only present in WT culture supernatant. Anything above the dotted horizontal line has a *P*-value of *P* < 0.01 and anything outside the two vertical dotted lines has a fold-change > 2×. Known and putative T2SS substrates are indicated in blue, and all proteins that are significantly affected when the PDZ domain is deleted are labeled.

To further verify the reliance of serine proteases on the PDZ domain for secretion and to assess if there are additional differences in the secretion profile (secretome) of the *epsC*∆*PDZ* strain, we subjected the culture supernatants of the WT and *epsC*∆*PDZ* mutant of *V. cholerae* to proteomic analysis. We performed liquid chromatography and tandem mass spectrometry (LC-MS/MS) of protein precipitates from overnight culture supernatants. Through quantitative spectral counting, we observed that five proteins were significantly (*P* < 0.01) reduced in abundance in the supernatant fractions of the *epsC*∆*PDZ* mutant (Table S1). The periplasmic quorum-sensing protein LuxP (43), the outer membrane-localized LPS assembly protein LptE (44), and two hypothetical periplasmic ABC-type transporter components VC_A0212 and VC_2622 were among the reduced proteins, but each was close to the 2× fold change cutoff. Only one of the proteins significantly reduced in abundance in the *epsC∆PDZ* supernatant was a previously identified T2SS substrate: serine protease VesB (Fig. 3; Table 1). Of the three homologous Ves proteases secreted by the T2SS (VesA, VesB, and VesC), VesB is responsible for ~80% of the serine protease activity observed in culture supernatants, which is consistent with our finding that extracellular serine protease activity is significantly reduced in the *epsC∆pdz* background (Fig. 2B) (7, 42).

**TABLE 1:** T2SS substrates detected in culture supernatants via quantitative LC-MS/MS

		N16961^*a*^			N16961 epsC∆PDZ^*a*^				
T2SS Protein	MW	1	2	3	1	2	3	Fold change (WT/∆PDZ)	*P*-value
VesB	43 kDa	0.00118	0.00094572	0.001183	6.23E-05	3.55E-05	0.000021406	28	0.00018
VesA	36 kDa	0.00078	0.0004687	0.001076	0.000315	0.000154	0	5	0.035
RbmC	104 kDa	0.00905	0.0080112	0.005876	0.005771	0.004548	0.0029777	1.7	0.06
Xds	94 kDa	0.01941	0.018573	0.001933	0.003306	0.001393	0.00024676	8.1	0.11
Tarp	150 kDa	0.0056	0.0051443	0.003875	0.004612	0.002588	0.0012618	1.7	0.14
Bap1	75 kDa	0.00386	0.00156	0.00491	0.00294	0.0012	0.00115	2	0.22
RbmA	30 kDa	0.00052	0.000406	0.00211	0.000511	0.000348	0.000063	3.3	0.28
CtxB	14 kDa	0.00067	0.00079937	0.000354	0.000513	0.000423	0.00039898	1.4	0.3
Lap	52 kDa	0.0081	0.010947	0.001571	0.006186	0.003753	0.00060536	2	0.35
VesC	60 kDa	0.01046	0.010306	0.006104	0.009989	0.006919	0.0022669	1.4	0.39
VC_A0583	30 kDa	0.001	0.0011593	0.000202	0.000758	0.000513	0.000028731	1.8	0.39
VC_1280	48 kDa	0.00171	0.0019317	0.002003	0.002311	0.000861	0.0012866	1.3	0.42
HapA	66 kDa	0.00367	0.0017431	0.002011	0.003257	0.000591	0.0010254	1.5	0.45
VC_A0140	44 kDa	0.0092	0.0045392	0.00344	0.007102	0.002711	0.0013208	1.5	0.46
VC_2298	22 kDa	0.0008	0.0019173	0.0004	0.000725	0.000619	0.00079634	1.5	0.52
HlyA	82 kDa	0.00316	0.001734	0.004813	0.004393	0.001812	0.00057763	1.4	0.53
VC_A0738	45 kDa	0.00095	0.0012192	0.000607	0.001162	0.000728	0.00022624	1.3	0.54
PrtV	102 kDa	0.00477	0.0046	0.0019	0.00437	0.00337	0.0008	1.3	0.55
GbpA	54 kDa	0.00153	0.00080982	0.002443	0.001569	0.00067	0.0015112	1.3	0.57
LapX	55 kDa	0.00869	0.01362	0.00181	0.010833	0.005817	0.00072233	1.4	0.64
NanH	86 kDa	0.02524	0.024544	0.003333	0.025298	0.013385	0.0039646	1.2	0.73
ChiA1	89 kDa	0.00396	0.00019912	2.22E-05	0.002719	0	0	1.5	0.77
VC_0769	63 kDa	0.00594	0.00023549	3.27E-05	0.004123	1.25E-05	0.000015029	1.5	0.79
ChiA2	90 kDa	0.01085	0	0	0.009815	0.000154	0	1.1	0.95

^*a*^ Normalized Spectral Abundance Factor (NSAF) for each pooled sample (pool “1,” “2,” or “3”).

Cholera toxin (CT) expression is low in LB medium, with only its B subunit (CtxB) detected by mass spectrometry (Table 1). Therefore, to better assess whether the PDZ domain removal affects CT secretion, we grew the WT and *epsC∆pdz* strains in AKI media to induce CT expression as previously reported (45), PRMM-precipitated proteins from the culture supernatants, and performed Western blots with anti-CT antibodies that recognize both the B and A subunits. We confirmed that CT is still secreted in the *epsC∆pdz* strain; however, the CT subunit A (CtxA) is no longer cleaved (Fig. S3). CtxA cleavage is a prerequisite for the generation of active CtxA-1 and the induction of secretory diarrhea caused by *V. cholerae* (46). T2SS substrates HapA, VesA, and VesB have each demonstrated the ability to cleave CtxA (7, 47, 48). It is important to acknowledge that *V. cholerae* strain N16961 is an El Tor strain with a *hapR* frameshift mutation that causes significant reductions in HapA expression (49, 50). Consistent with this, a mutant N16961 with *hapA* deleted from the genome shows no change in CtxA cleavage compared to WT N16961 (Fig. S3). On the other hand, the deletion of VesA or VesB leads to a reduction of CtxA cleavage, as is shown here and previously reported for these same strains (7). The loss of CtxA processing in the *epsC∆pdz* mutant is consistent with the reduced secretion of VesB.

### Validation of the PDZ-dependence of VesB secretion through the T2SS

**Fig 4:**
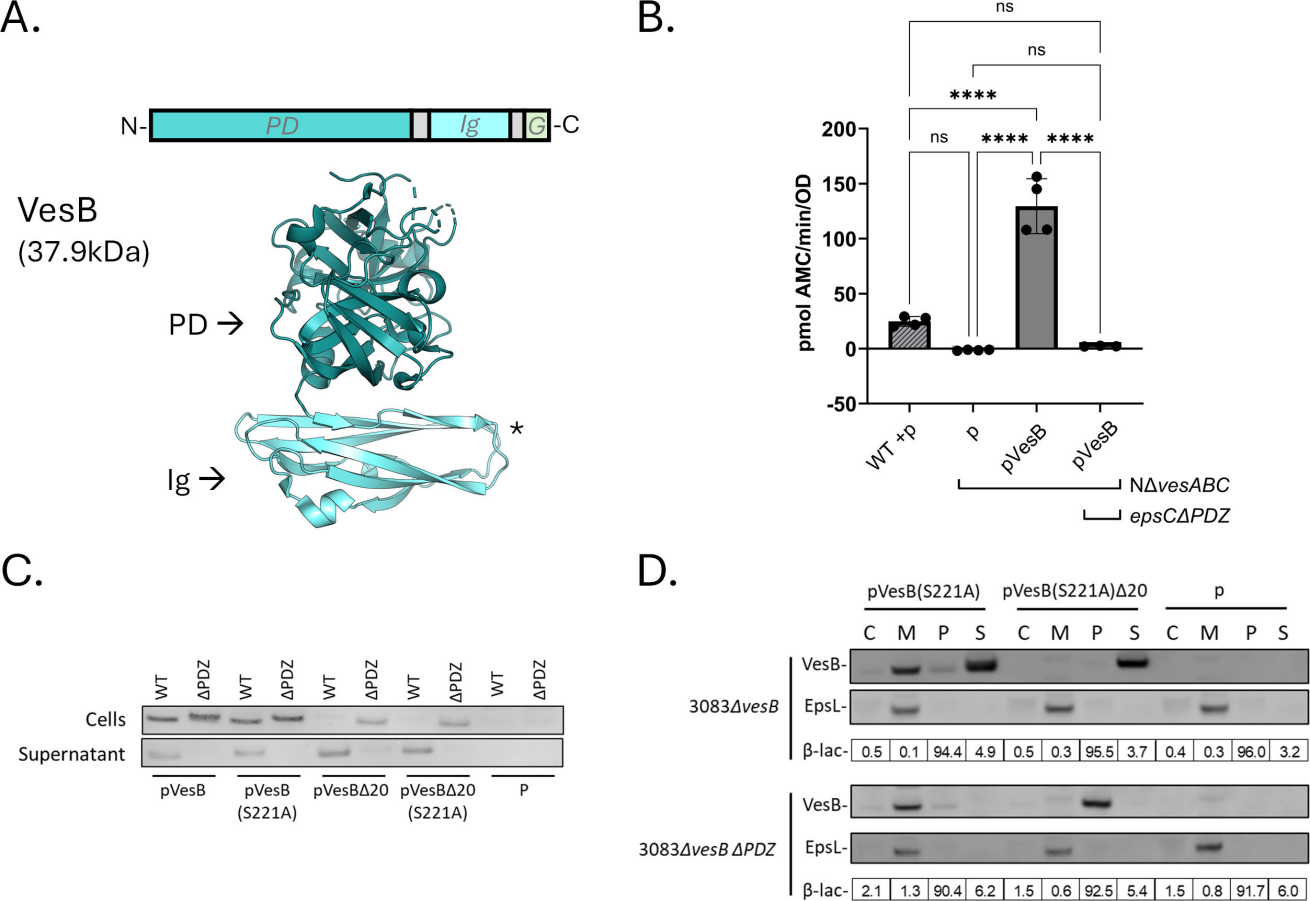
VesB secretion and activity are reduced in the *epsC∆PDZ* mutant. (**A**) Schematic diagram and crystal structure of truncated VesB lacking the signal peptide and GlyGly-CTERM (PDB: 4LK4) with the protease domain (PD) and Ig-fold (Ig) indicated. The asterisk indicates where the GlyGly-CTERM (G) would be located. (**B**) VesB activity in culture supernatants measured with a methylcoumarin-conjugated peptide (Boc-Gln-Ala-Arg-AMC). Strains containing an empty vector are indicated by “p,” and data represent the mean +/− standard deviation of biological replicates each measured in technical triplicate (*n* ≥ 3). Ordinary one-way ANOVA with a Tukey correction for multiple comparisons was used for analysis (ns = not significant, ****P ≤ 0.0001). (**C**) Representative Western blot of three biological replicates of *V. cholerae* cultures with ectopic expression of active (VesB), inactive (VesB-S221A), and truncated (VesB∆20) VesB constructs along with an empty vector control (P). Detection was performed with rabbit anti-serum to VesB and goat anti-rabbit HRP. “WT” and “∆PDZ” refer to the status of chromosomal *epsC*. (**D**) Paired VesB and EpsL (membrane fraction control) Western blots representative of three biological replicates after fractionation via polymyxin B treatment, sonication, and ultracentrifugation. The inner/outer membrane fraction (M), cytoplasmic fraction (C), periplasmic fraction (P), and culture supernatant (S) are indicated. All samples were analyzed for β-lactamase activity in technical triplicate (periplasmic control), and the activity for each fraction is shown as a percentage of the total activity.

VesB is a trypsin-like serine protease with an immunoglobulin (Ig)-like domain and a C-terminal transmembrane helix called GlyGly-CTERM, which is required for association with the cell surface following C-terminal processing and secretion (Fig. 4A) (42, 51). To validate our LC-MS/MS findings for the secretion of VesB, we ectopically expressed *vesB* in a *∆vesABC* or *∆vesABC* epsC*∆PDZ* mutant and found that PDZ deletion entirely abolished protease activity in the supernatant (Fig. 4B). We also confirmed this finding via Western blot analysis using polyclonal VesB antiserum of LB culture samples from ∆*vesB* mutants expressing ectopic VesB in the absence or presence of the PDZ domain of EpsC (Fig. 4C). Plasmid expression of different VesB constructs clearly showed that the PDZ-dependent secretion of VesB is not contingent on its proteolytic activity (VesB-S221A is catalytically inactive) and that VesB lacking residues C-terminal of the GlyGly motif (VesB∆20) is still secreted and PDZ-dependent (Fig. 4C) (42, 51). Since VesB is a zymogen that is activated after it is secreted, the effect on VesB activity is also apparent in immunoblots where VesB expressed in an *epsC*∆*PDZ* background does not appear in the same location of the SDS-polyacrylamide gel, instead appearing slightly higher due to the presence of its propeptide.

**Fig 5:**
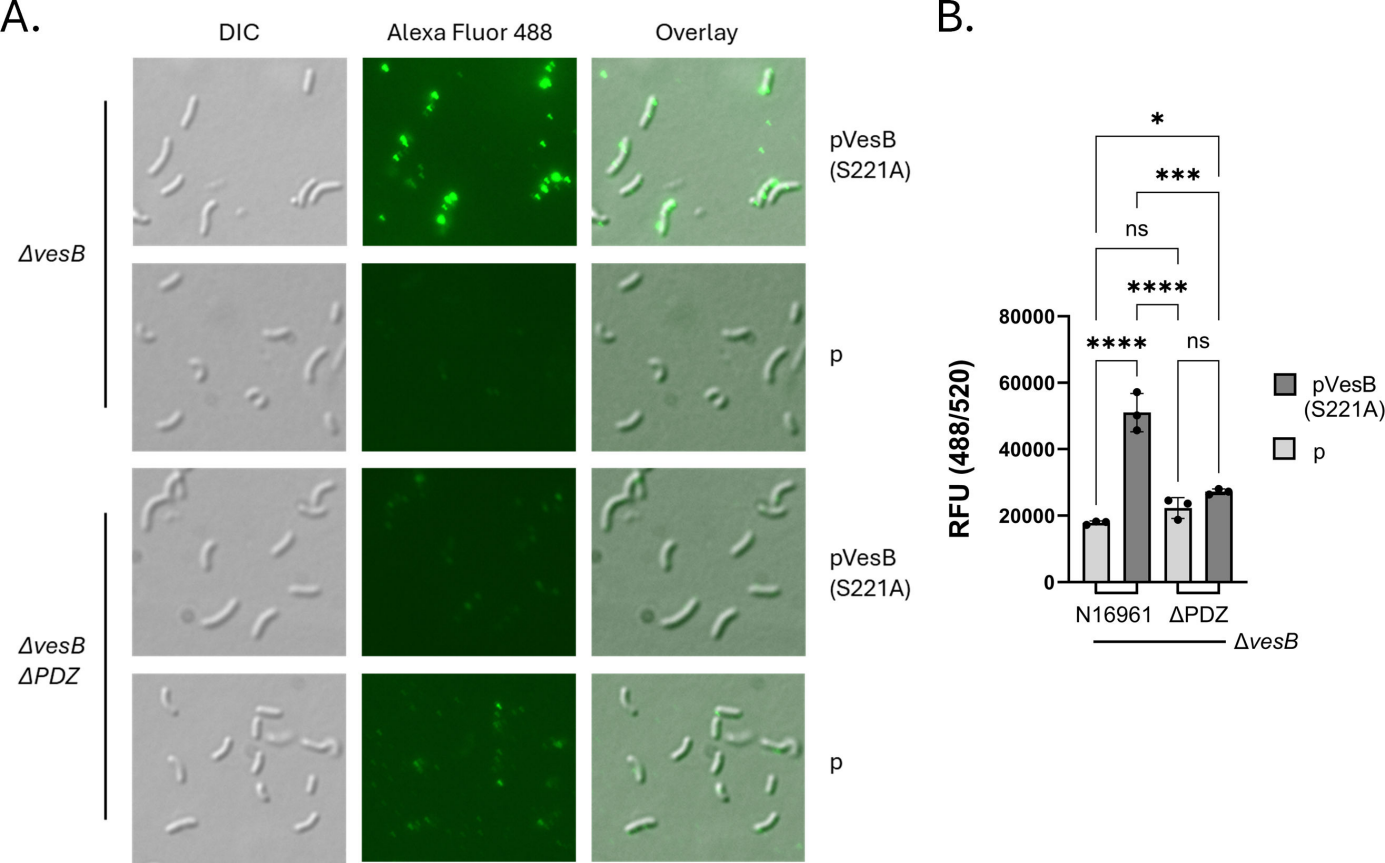
VesB surface localization is reduced in the *epsC∆PDZ* mutant. (**A**) Immunofluorescence microscopy using rabbit anti-VesB antiserum and goat anti-rabbit secondary conjugated to Alexa Fluor 488. Matching images of differential interference contrast (DIC) and Alea Fluor 488 detection are both shown along with an overlayed image depicting *V. cholerae* ∆*vesB or* ∆*vesB epsC∆PDZ* mutant strains expressing ectopic *vesB(S221A*) or containing empty vector (**P**) controls. Representative of five images taken from three biological replicates each. (**B**) Quantification of VesB surface localization measured in a black 96-well plate with relative fluorescence units (RFU) reported from three biological replicates in technical duplicates. Ordinary one-way ANOVA with Tukey test for multiple comparisons was used for analysis (ns = not significant, *P ≤ 0.05, ***P ≤ 0.001, ****P ≤ 0.0001).

We have previously shown that WT VesB is predominantly localized to the cell surface with a limited amount released to the extracellular space following transport by the T2SS (52, 53). To determine whether VesB surface localization is affected by the removal of PDZ from EpsC, we used VesB anti-serum along with an AlexaFluor 488 conjugated secondary antibody to detect surface-localized VesB on intact cells (52). WT and *epsC∆PDZ V. cholerae* strains lacking chromosomal *vesB* (∆*vesB*) were grown for 3–4 h with IPTG induction of plasmid-encoded, catalytically inactive VesB (VesB-S221A), labeled, and analyzed by immunofluorescence microscopy or total fluorescence of suspended cells (Fig. 5A and B) (52, 53). These experiments demonstrate that, in contrast to VesB expressed in the WT strain, VesB is not detected on the surface of *epsC*∆*PDZ* mutant cells.

Subcellular fractionation with polymyxin B to release periplasmic content, followed by sonication and ultracentrifugation (54), confirms that VesB is associated with total membrane and culture supernatant fractions in WT *V. cholerae*, while VesB∆20 is entirely in the culture supernatant (Fig. 4D). Western blot of the inner membrane T2SS component EpsL was used as a fractionation control for the membrane fraction, and the activity of periplasmic β-lactamase expressed from the pMMB67EH plasmid was used as a control for the periplasmic fraction. These data are consistent with our previous findings, emphasizing the required role of the GlyGly-CTERM in VesB surface localization, and the fact that VesB is still properly secreted without it (53). In the *epsC*∆*PDZ* mutant, VesB is entirely associated with the total membrane fraction and VesB∆20 is localized to the periplasmic compartment (Fig. 4D). Taken together with the immunofluorescence microscopy, these data demonstrate that outer membrane translocation of VesB (a pre-requisite for surface localization) is fully dependent on PDZ.

### Secretion of VesB homologs is only partially affected by PDZ deletion

**Fig 6:**
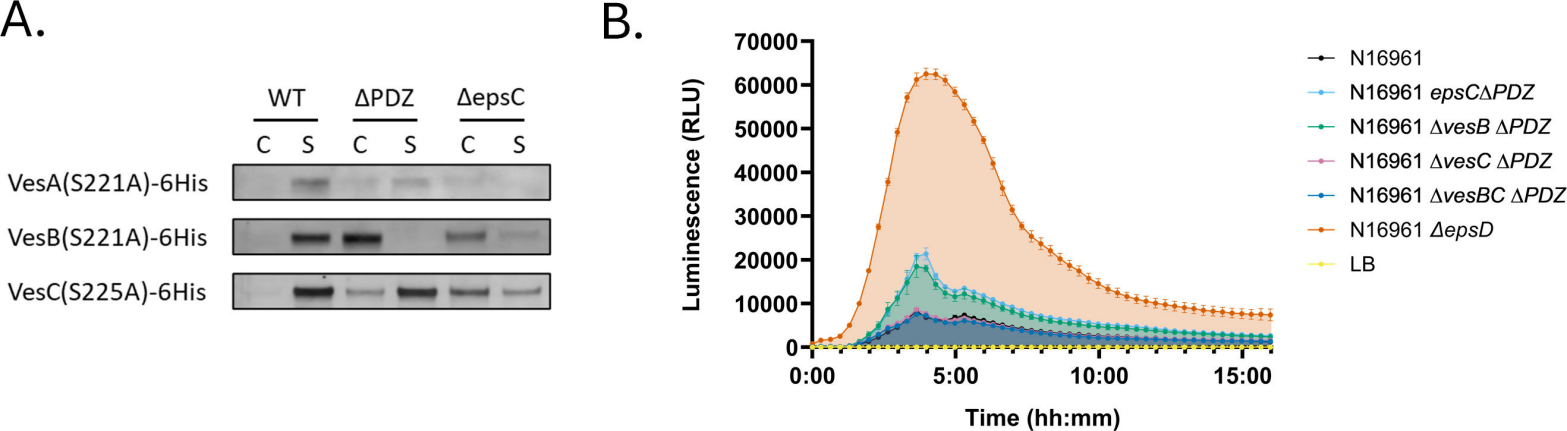
Secretion of the Ves proteases is differentially affected by the *epsC∆PDZ* deletion. (**A**) Western blots representative of three biological replicates showing the secretion of His-tagged Ves proteases. “WT” refers to the status of chromosomal *epsC*, “C” is the cell fraction, and “S” is the culture supernatant. (**B**) Luminescence was recorded as a measure of extracytoplasmic stress during the growth of the indicated *V. cholerae* strains ectopically expressing the bacterial Lux reporter from the *V. cholerae* RpoE promoter. Relative luminescence units (RLU) over time are depicted as the mean +/− standard deviation for two biological replicates measured in technical triplicate.

Unequal reliance of T2SS substrates on PDZ for secretion has been observed among pectate lyases in *Dickeya dadantii* (39). VesB has two homologs in *V. cholerae*, serine proteases VesA and VesC, and LC-MS/MS data indicate that neither VesA nor VesC secretion is significantly affected by deletion of the PDZ domain (Table 1). To verify this finding, we created His-tagged versions of each of the Ves proteases by replacing the residues C-terminal of the GlyGly motif with a His_6_ tag and examined their secretion using Western blotting. Unlike VesB, both VesA and VesC are still mostly secreted in the *epsC*∆*PDZ* mutant, with some minor retention in the cell fraction (Fig. 6A). Intracellular retention of VesC in T2SS mutants is toxic for *V. cholerae,* and some T2SS mutants contain mutations in *vesC* that may partially relieve the lethal phenotype of T2SS inactivation (32). If we monitor extracytoplasmic stress using the RpoE promoter fused to a bacterial Lux reporter gene as previously described (55), the low level of VesC retention observed in the *epsC*∆*PDZ* mutant correlates with a slight increase in extracytoplasmic stress. However, the level of stress is much higher when the entire *eps* operon or *epsD* is deleted, as previously demonstrated (Fig. 6B) (34). When the *vesC* gene is deleted in the *epsC*∆*PDZ* mutant, the stress is relieved. Despite the slight stress with minor VesC retention in the *epsC*∆*PDZ* mutant, it is important to note that T2SS mutants still have a growth defect when *vesC* is deleted (Fig. S4). This finding aligns with previous reports suggesting that VesC retention is not the sole cause of the severe growth defects of *V. cholerae* mutants with an inactivated T2SS (32). Together, these data demonstrate that the three serine protease homologues secreted through the *V. cholerae* T2SS are differentially affected by deletion of the PDZ domain. VesB requires the PDZ domain for secretion, whereas VesC and VesA are still secreted in the absence of PDZ, albeit slightly less efficiently.

### The VesB Ig-fold contains a secretion signal that is PDZ-dependent

**Fig 7:**
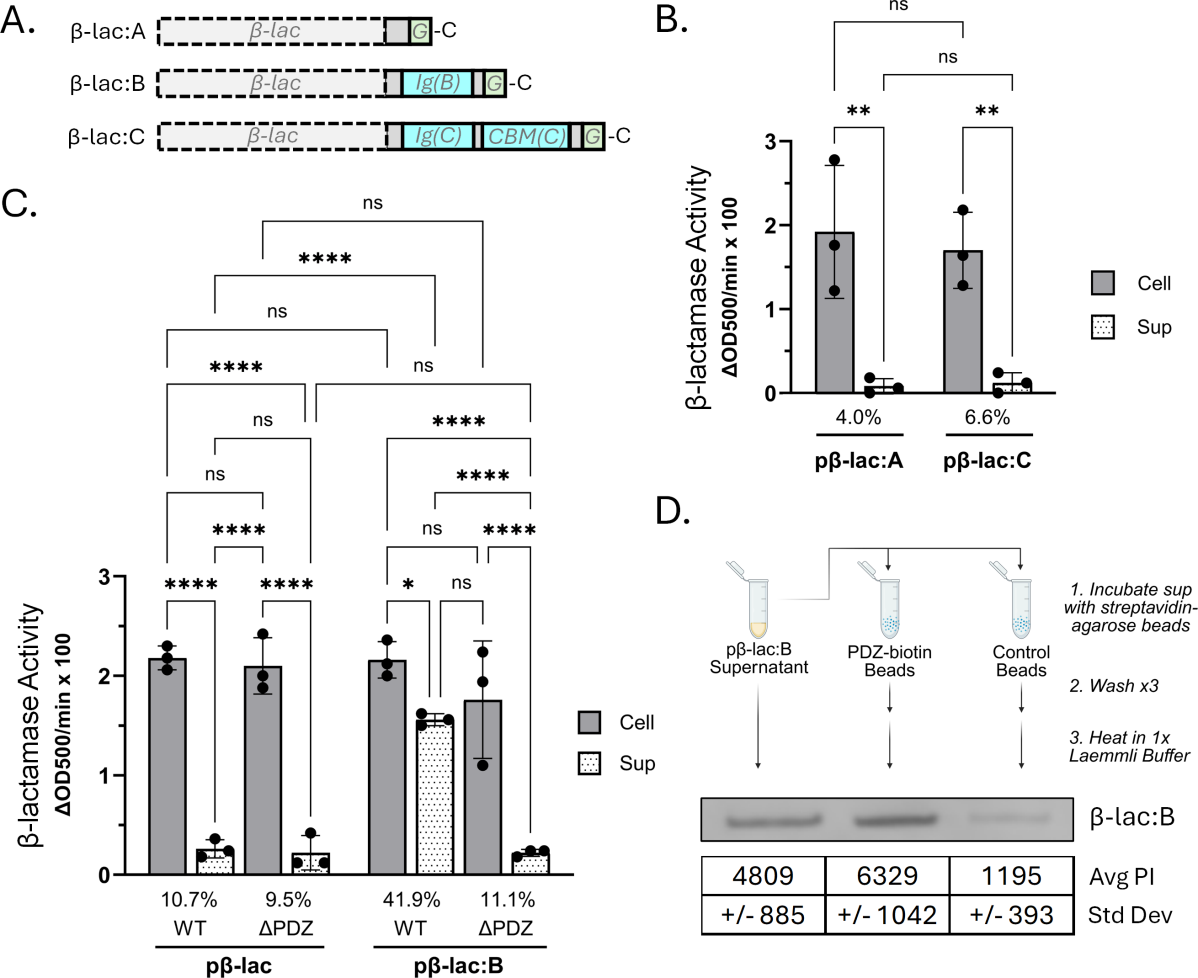
The VesB Ig-fold enables β-lactamase secretion in a PDZ-dependent manner. (**A**) Schematic diagrams depicting three chimeric constructs with β-lactamase replacing the protease domain of VesA (β-lac:A), VesB (β-lac:B), or VesC (β-lac:C). (**B**) β-lac:A or β-lac:C were expressed in WT *V. cholerae* and β-lactamase activity was measured in culture supernatants as well as sonication-disrupted cells from three biological replicates measured in technical triplicate with nitrocefin as a substrate. Activity is represented by the change in OD 500 nm per minute normalized to the OD_600_ of the original culture, with the average background activity (empty vector control) subtracted from each sample. Below each cell/supernatant pair is the percentage of activity that belongs to the supernatant fraction as a portion of the total activity of the cell and supernatant from that culture. (**C**) Activity of native β-lactamase and β-lac:B expressed in either WT or *epsC∆PDZ V. cholerae* strains measured as in (**B**) (*n* = 3). All final values were multiplied by 100 for clarity, and a two-way ANOVA with Tukey‘s correction for multiple comparisons was performed (ns = not significant, *P ≤ 0.05, **P ≤ 0.01, ***P ≤ 0.001, ****P ≤ 0.0001). (**D**) Methodological diagram (created using BioRender) of a pulldown performed with streptavidin-agarose beads without (control beads) or with bound biotinylated PDZ (PDZ-biotin beads). Supernatants from three different overnight *V. cholerae* cultures expressing β-lac:B were used for the pulldowns and a representative Western blot is shown along with the average pixel intensity (Avg PI) and standard deviation as measured with ImageJ.

A study by Pineau et al. demonstrated that the fibronectin (Fn3) domain of the T2SS substrate PelI interacts directly with the PDZ domain of EpsC homologue OutC in *D. dadantii* (40). Since the Fn3 domain is a subclass of the Ig superfamily, we decided to test if the Ig-fold domain of VesB plays a role in secretion and in VesB’s reliance on the PDZ domain. Deleting the Ig-fold domain of His-tagged VesB∆20 led to an unstable protein that was not detected with anti-His-tag antibodies nor with VesB antibodies (Fig. S5). Due to the deleterious effect on VesB stability when its Ig-fold domain is removed, we attempted an alternative strategy. We replaced the protease domain of VesB, VesA, and VesC with β-lactamase (a non-homologous periplasmic enzyme), creating three different β-lactamase chimeras, β-lac:B, β-lac:A, and β-lac:C (Fig. 7A) and assessed if they could be secreted through the T2SS. We monitored secretion by measuring β-lactamase activity in the supernatant and in sonication-disrupted cells using nitrocefin as a substrate (Fig. 7B and C). We observed that while only a small amount (approximately 5%) of active β-lac:A and β-lac:C were detected in the culture supernatant (Fig. 7B) similar to native β-lactamase (Fig. 7C), the amount of active β-lac:B was significantly higher (42%; Fig. 7C). Importantly, this increased supernatant activity of β-lac:B was not observed in the *epsC∆PDZ* mutant, suggesting that the Ig-fold of VesB is capable of directing β-lactamase to the extracellular environment in a PDZ-dependent manner.

To determine if the Ig-fold of VesB interacts with the PDZ domain, we performed a pulldown experiment of β-lac:B using purified PDZ. To this end, we N-terminally AVI-tagged (56, 57) and purified the PDZ domain of EpsC, biotinylated the tag with purified BirA, and bound the biotinylated PDZ to Streptavidin-agarose resin. When supernatants from overnight cultures of *V. cholerae* expressing β-lac:B were incubated with the PDZ-bound beads or with control beads lacking PDZ, a much higher amount of β-lac:B was captured by the beads bound to PDZ (Fig. 7D). These data suggest that there is a bona-fide PDZ-dependent secretion signal in the Ig-fold domain of VesB and that secretion of the chimeric β-lac:B construct may be facilitated by direct interaction with the PDZ domain of EpsC.

## DISCUSSION

Previous studies have demonstrated that deletion or inactivation of T2SS components (*epsD, epsE,* etc*.*) leads to poor growth, extracytoplasmic stress, membrane leakiness, and selection of suppressor mutations in *V. cholerae* (32–34). Here, we demonstrate that the expression of EpsC∆HR does not complement the growth defect of an *epsC::kan* mutant, nor does it restore serine protease secretion (Fig. 1C and 2A). However, the growth rate of the *epsC::kan* mutant expressing EpsC∆PDZ is indistinguishable from WT *V. cholerae* despite being unable to complement serine protease activity in the supernatant (Fig. 1C and 2A). This appears to be due to the limited impact of the PDZ domain deletion on T2SS function in *V. cholerae*, with PDZ removal only significantly affecting the secretion of one T2SS substrate, the serine protease VesB. Questions remain as to why more dramatic disruptions of the *V. cholerae* T2SS leads to the aforementioned phenotype. The most likely explanation for the growth defect of *eps* mutants is that T2SS inactivation causes the retention of secreted enzymes in the periplasm and their off-target activity leads to cytotoxicity. VesC is an obvious candidate for this phenotype since it has previously been shown that the gene has inactivating mutations in several *eps* mutants (32). However, our findings demonstrate that while even a small amount of VesC retention does lead to induction of the RpoE stress response in the *epsC∆PDZ* mutant, VesC is not the sole cause of growth defects in T2SS mutants. Our data demonstrate that the ∆*eps* mutant that lacks the entire *epsC-epsN* operon still grows poorly when the *vesC* gene is deleted (Fig. S2A). It is possible that the phenotype of *V. cholerae* T2SS mutants may be the result of compounding issues from the periplasmic retention of enzymes like VesC and perhaps also the loss of enzyme function extracellularly.

Using quantitative mass spectrometry, we demonstrated that VesB is the only T2SS substrate significantly affected by PDZ deletion among the nearly two dozen substrates in *V. cholerae* (Table 1). Subsequent immunoblotting analysis of VesB and its homologs confirmed the dependence of VesB’s secretion on the PDZ domain and showed that the secretion of VesA and VesC is only partially affected when EpsC is expressed without its PDZ domain (Fig. 5A). Previous work showed differential effects on the secretion of pectate lyase homologs in *Dickeya dadantii* (39), and our data describe a strikingly similar phenomenon with the Ves proteases in *V. cholerae*. These data contribute to a growing body of evidence that the C-terminal region of EpsC and its homologs is an important component of the T2SS that is required for the secretion of some, but not all, T2SS substrates. Unexpectedly, the abundance of four additional proteins (LuxP, LptE, VCA0212, and VC2622) was also reduced in the supernatant of the *epsC*∆PDZ mutant. Since each of these proteins is confirmed or predicted to localize to the periplasmic compartment or inner leaflet of the outer membrane, the small amount of these proteins in the WT culture supernatant may be due to their presence in outer membrane vesicles (OMVs), which were not removed in our study. Their reduced abundance in the supernatant of the *epsC*∆PDZ mutant may be due to proteolytic degradation by periplasmically retained serine protease VesB, resulting in lower levels present in the OMVs. Alternatively, their expression may be reduced due to the low level of stress observed in the *epsC*∆PDZ mutant.

The data presented here demonstrate that the periplasmic protein β-lactamase can be secreted in a PDZ-dependent manner from *V. cholerae* when it is fused to the VesB Ig-fold domain (β-lac:B). Additionally, biotinylated PDZ bound to streptavidin-conjugated agarose beads can capture the secreted β-lac:B chimera present in the culture supernatant, demonstrating that the VesB Ig-fold contains structural information important for PDZ-dependent secretion and suggesting that direct interaction with PDZ may be involved. Ig-fold and Ig-like domains containing essential secretion information have also been identified in T2SS substrates from other species. For instance, pullulanase (PulA) is a well-characterized T2SS substrate in *K. pneumoniae* and *K. oxytoca* (58–60) that has an N-terminal domain comprised of seven antiparallel β-strands folded into two β-sheets resembling an Ig-fold domain. While it remains to be determined whether PulA requires the PDZ domain of EpsC homologue PulC for secretion, the Ig-fold region of PulA appears to be partially responsible for recognition by the *K. oxytoca* T2SS because disruption of this domain dramatically reduces PulA secretion (61). Relatedly, a disordered loop in the Fn3 domain (a member of the Ig domain superfamily) of pectate lyase PelI in *D. dadantii* has been shown to interact directly with the PDZ domain of the EpsC homolog OutC (40). PelI itself is only partially affected by the PDZ domain deletion, with 50%–80% still secreted, suggesting that direct interaction with PDZ may increase the secretion efficiency of some substrates (39).

To better understand how substrates are recognized by the T2SS, we and others have employed the strategy of making protein chimeras. Early investigations of *K. pneumoniae* PulA secretion involved generating PulA chimeras with periplasmic proteins like β-lactamase or non-homologous T2SS substrates from other species (61, 62). Other researchers have taken the approach of identifying homologous T2SS substrates from different species (each secreted only by their cognate T2SS) and testing the secretion of protein chimeras with unique structural characteristics swapped between the two (40, 41, 63). What this work and the work of others have revealed is that each T2SS substrate contains one or more structural features, distributed throughout the protein, that likely engage with different components of the T2SS along the path of secretion. Whether the regions identified are more important for initial recognition and recruitment to the T2SS or are involved in engagement and coordination of the piston-like secretion process remains to be determined. Intuitively, it has been assumed that the exterior parts of the system, such as EpsC and EpsD, are more likely to be involved in recruitment, while compatible transient interactions between substrates and the components of the T2SS luminal space—the endopilus tip and EpsD interior—must follow for efficient secretion. Importantly, the diversity of T2SS substrates and lack of a linear “universal secretion signal” indicate that the information recognized by the T2SS is structural and possibly variable, even within the substrate repertoire of a single species. We speculate that different components of the T2SS play relatively greater or lesser roles in the engagement of different substrates, with the C-terminal region of EpsC and its homologs being the clearest example of this.

Currently, it is unclear whether the C-terminal domain of EpsC and its homologs function as an adaptor to meet the demands of secreting such a diverse repertoire of protein substrates while excluding other periplasmic proteins, or if the domain is an ancestral vestige that other substrates used to rely upon but have since evolved away from. The argument that it allows the T2SS to diversify its secretion repertoire seems more likely, considering that most T2SSs have either a PDZ or coiled-coil domain. *L. pneumophila* is an exception with its truncated EpsC homolog more akin to the EpsC counterpart in the Type-IV pilus (38), which may indicate a system that has either adapted fully away from the need for a PDZ domain or is a system with a repertoire of substrates with more common features. We contend that the reason for differential reliance on PDZ may vary for each species, indicating something more broadly about the T2SS. This may simply be the inevitable evolutionary result of a complex system adapting to the secretion of many structurally diverse substrates over time.

## MATERIALS AND METHODS

### Bacterial strains, cloning, and construct creation

All primers and strains used in this study are listed in Tables S2 and S3, respectively. *V. cholerae* El Tor 01 strains N16961 (Inaba subtype) and 3083 (Ogawa subtype) were used throughout this work, along with the N16961 ∆*vesA,* ∆*vesB,* ∆*vesC,* ∆*vesAB,* and ∆*vesABC* strains previously published (7). Gene disruption was performed, as previously reported, by homologous recombination with a suicide vector (pCVD442) containing ~500 bp of homology on both ends of a genomic region to be deleted, which was followed by sucrose selection and PCR screening for the modification of interest (33).

Plasmid constructs were created by overlap extension PCR, followed by restriction enzyme digestion and cloning of the resultant DNA insert into the target plasmid (pCR-Script, pMMB67EH, etc.) using T4 DNA ligase. Plasmids were conjugated into different *V. cholerae* strains using triparental conjugation including the *E. coli* MM294 helper strain carrying pRK2013 plasmid and *E. coli* strain MC1061 containing the plasmid of interest (pMMB67 or pBBR) (33).

The DNA fragment corresponding to the PDZ domain of EspC was PCR-amplified from *V. cholerae* genomic DNA using primers PDZ_bio_Nco and PDZ_Hind. The resulting DNA fragment was digested with NcoI and HindIII and ligated into the pRSF-NT vector (64), creating the pRSF-bioPDZ plasmid for the expression of the PDZ domain with AviTag for biotinylation. The *birA* gene was PCR-amplified from *E. coli* genomic DNA using primers birA_Nco and birA_Hind. The DNA fragment was digested with NcoI and HindIII and ligated into pRSF-NT, creating pKV1622 plasmid for expression of BirA biotin ligase with a His_6_-tag.

### Protein precipitation and LC-MS/MS of culture supernatants

Wild-type (WT) *V. cholerae* N16961 and N16961*epsC∆PDZ* were grown at 37°C for 16 h in Luria-Bertani broth (LB). Culture supernatants were isolated via centrifugation and filter sterilized before being subjected to protein precipitation with an equal volume of pyrogallol red-molybdate-methanol (PRMM) solution (65). The mixture was incubated for 2 h at ambient temperature, followed by a 16 h incubation at 4°C as described previously (7). Precipitated proteins were pelleted at 10,000 × *g* for 1 h at 4°C, washed with cold acetone, and centrifuged twice before air-drying. Protein pellets were resuspended in 1× Laemmli sample buffer and heated for 20 min at 90°C. Three pools of four samples each from the WT and *epsC∆PDZ* mutant strain were normalized by the optical density (OD_600_ nm) of their originating culture and analyzed by SDS-PAGE and Coomassie staining before further correction with 1× Laemmli sample buffer based on the pixel density of the lanes as measured by ImageJ.

Normalized samples were subjected to GeLC10-tandem mass spectrometry (LC-MS/MS) and label-free quantitation as previously described (52), but with modifications. For this analysis, we used a Waters M-Class HPLC system coupled with a Fusion Lumos mass spectrometer (Fisher). The trapping columns were packed with Luna C18 resin (Phenomenex), and the Orbitrap was run with a 3 s Cycle for MS and MS/MS. Normalized Spectral Abundance Factor (NSAF) was used to compare the three pooled samples of WT culture supernatants to those of *epsC∆PDZ* pooled samples. Scaffold 5 software was used to collate, filter, and analyze the data. The complete mass spectrometry data set (PXD062995) was deposited to the ProteomeXchange Consortium (http://proteomecentral.proteomexchange.org).

### Protease activity

Protease activity was measured as previously described (42, 66). Briefly, cell or culture supernatant fractions were mixed with Boc-Gln-Ala-Arg-7-amino4-methylcoumarin (Boc-QAR-MCA) to a final concentration of 50 µM in HEPES buffer (25 µM, pH 7.5) with a total reaction volume of 100 µL, and the generation of liberated methylcoumarin (MCA) was measured at Ex380nm/Em440nm every 70 s over 15 min at 37°C using an H1 Synergy plate reader (Biotek). The rate of cleaved MCA in pmol/min was determined with a standard curve of known MCA concentrations, and the final rate was subsequently normalized by the OD_600_ of the original culture. ≥3 biological replicates, each tested in triplicate, were analyzed for each condition.

### Western blots

Cell or culture supernatant fractions were normalized by OD_600_ and subjected to SDS-PAGE on 4-12% Bis-Tris gradient gels and semi-dry transfer to nitrocellulose using the Transblot Turbo platform. Membranes were blocked in Tris-buffered saline with 0.05% TWEEN20 (TBST) + 10% skim milk for at least 2 h with rocking, washed three times in TBST for 5 min each, and incubated either 2 h at ambient temperature or overnight at 4°C with rocking in TBST containing polyclonal rabbit antibody (anti-VesB or anti-EpsL) diluted 1:5,000–1:20,000 or rabbit anti-6His antibody (Invitrogen) diluted 1:5,000. After washing, blots were incubated in TBST containing a 1:5,000 dilution of goat anti-rabbit HRP antibody (Bio-Rad) for 1 h at ambient temperature, washed in TBST, developed with Pierce ECL2 chemiluminescence substrate, and detected on the Amersham Typhoon platform with Cy2 settings at 375 V.

### Growth analysis

Stationary-phase cultures in LB from overnight growth at 37°C with agitation in the presence of appropriate antibiotics were diluted to an OD_600_ of 0.01 in fresh LB media with antibiotics. Samples were then loaded into microplates and analyzed for growth via OD_600_ at 37°C with agitation using a Bioscreen C Growth Curve analyzer.

### Beta-lactamase

Beta-lactamase activity was assessed by analyzing the hydrolysis of nitrocefin as previously described (67). Briefly, nitrocefin resuspended in PBS, and 5% DMSO was added to culture supernatants to a final concentration of 100 µM and the absorbance at 500 nm was recorded using an H1 Synergy plate reader. Sonication-disrupted cell fractions were tested using the same protocol.

### RpoE::Lux assays

To measure extracytoplasmic stress, *V. cholerae rpoE* promoter activity was assessed using a bacterial Lux system as previously reported (55). Briefly, a pBBR plasmid with the *V. cholerae rpoE* promoter positioned upstream of the *luxCDABE* (*lux*) cassette was used. Strains containing this reporter plasmid were grown overnight in LB with appropriate antibiotics at 37°C with shaking before diluting 1:100 into fresh media. Two hundred microliters of the dilution was loaded into a white 96-well plate and luminescence was measured over time at 37°C with agitation using a H1 Synergy plate reader.

### Cell surface detection of VesB and microscopy

Detection of VesB on the cell surface was carried out as previously described (52). Briefly, bacteria were grown to mid-log phase in LB medium with appropriate antibiotics before being washed and blocked with 2% bovine serum albumin (BSA) in Tris-buffered saline (TBS). VesB anti-serum that was pre-incubated with ∆*vesABC* cells to remove cross-reactive antibodies was then added (1:1,000), followed by washes and incubation with a 1:1,000 dilution of Alexa Fluor 488 Fab goat anti-rabbit IgG (Fisher). After a final wash, fluorescence of labeled bacteria was measured in a Biotek H1 Synergy plate reader (Ex 495 nm/Em 519 nm). In parallel, the bacteria were applied to 1% agarose pads and visualized with a Nikon Eclipse 90i fluorescence microscope with a 100× oil-immersion objective and differential interference contrast (DIC) or fluorescence microscopy with 100 ms exposure (Ex 488 nm/Em 525 nm). Images were captured with an attached CoolSNAPHQ digital camera. DIC images were processed in Adobe Photoshop, with brightness and contrast curve adjustments applied uniformly across all images. Fluorescence images were changed from grayscale to RGB color mode with input and output green curve adjustments applied uniformly across all images. The DIC and fluorescence images were overlaid as Smart Objects to generate the final merged images.

### PDZ-biotin purification

The pRSF-bioPDZ plasmid was transformed into *E. coli* T7 Express::pRARE2 (NEB, C2566) cells and a starter culture containing 2% glucose, 50 mg L^–1^ Kan, 15 mg L^–1^ Cm in LB was grown overnight. A 1:60 dilution of overnight culture was inoculated into 2.4 L of LB and bacteria were grown to an OD_600_ of 0.6 at 37°C before the addition of 400 µM isopropyl β‐d‐thiogalactopyranoside (IPTG) for induction at 16°C. After incubation for 12 h, the cultures were centrifuged at 4,000 × *g* for 20 min at 4°C. The cells were then resuspended in 20 mM Tris pH 8.0, 300 mM NaCl, and 10 mM imidazole buffer. Bacteria were lysed using two passes through a Microfluidizer (Microfluidics). Cell debris was removed via centrifugation at 14,000 × *g* for 1 h at 4°C. Protein was purified by passage over a Ni-affinity column containing His-Trap chelating resin (Cytiva). The column was washed with 20 mM Tris pH 8.0, 300 mM NaCl, 10 mM imidazole buffer. The protein was then eluted using the same buffer with 250 mM imidazole. The His-tag was removed by digestion with the TEV protease during overnight dialysis in 20 mM Tris, pH 8.0, 300 mM NaCl buffer. The protein was passed via a Ni-affinity column to remove the His_6_-tag and TEV protease. After assaying eluates for purity by SDS-PAGE, fractions containing AviTag-PDZ protein were concentrated using a Amicon Ultra 4 with a 3K MWCO (Millipore) and then buffer exchanged into a final storage buffer of 50 mM Tris pH 8.5, 100 mM NaCl using continuous dilutions in the spin concentrator followed by supplementation with 25% glycerol and flash frozen in liquid nitrogen. BirA biotin ligase was expressed and purified similarly to AviTag-PDZ, except the His_6_-tag was not removed.

Biotinylation of the AviTag-PDZ was accomplished using the purified BirA enzyme *in vitro*. Briefly, 100 µM of AviTag-PDZ protein in 952 µL of PBS was combined with 5 µL of 1 M MgCl_2_, 20 µL of 100 mM ATP, 20 µL of 50 µM BirA, and 5 µL of 50 mM d-biotin. Samples were incubated for 1 h at 30°C with gentle mixing on a rocking platform. An additional 20 µL of 50 µM BirA and 5 µL of 50 mM d-biotin were added, and the reaction was allowed to progress for an additional hour. His_6_-BirA was then removed by incubating for 30 min at room temperature with a 50% slurry of 0.1 mL of HisTrap beads in PBS, followed by centrifugation and decanting from the beads. The sample was then dialyzed into PBS to remove excess biotin, flash-frozen in liquid nitrogen, and stored at –80°C.

### Agarose bead pulldown

Sixty microliters of High-capacity Streptavidin-agarose bead resin (Thermo Fisher, CAT: 20357) was washed with cold HEPES buffer (10 mM HEPES, 150 mM NaCl, pH 7.4) before resuspension in either 600 µL of buffer (control beads) or 600 µL of buffer containing 0.5 mg/mL of PDZ-biotin and rotated overnight at 4°C. The beads were then washed with cold buffer three times, resuspended in buffer containing 0.1% BSA, rotated at 4°C for 2 h, and washed again three times. Twenty microliters of blocked PDZ beads and 20 µL of control beads were resuspended in 400 µL of filtered culture supernatant from *V. cholerae* expressing β-lac:B. The beads were incubated for 1 h at 4°C with continuous rotation before being washed with cold buffer and finally resuspended in 40 µL 1× Laemmli buffer. The samples were heated to 99°C for 10 min, the beads pelleted, and 18 µL of the resulting supernatants were subjected to a 4%–12% Bis-Tris gradient SDS-PAGE and immunoblotting. A β-lactamase monoclonal mouse antibody (Invitrogen, MA-1 20370) along with an HRP-conjugated goat anti-mouse antibody (Invitrogen, PI31430) were used to detect β-lac:B. ImageJ (68) analysis of the pixel intensity of the resulting β-lac:B signal is shown as the mean +/− standard deviation of *n* = 3 experiments.

### Statistical analysis

All data were analyzed in GraphPad Prism 10 and are presented as mean ± standard deviation, with significance of the following statistical comparisons indicated. Differences between the two groups in the LC-MS/MS analysis were determined by a paired, two-tailed Student’s *t*-test. One-way ANOVA analysis was used to compare three or more related groups with a Tukey correction for multiple comparisons between all samples tested and a Dunnett’s test in Fig. 2A for comparing all samples to the 3083 *epsC::Kan* + p*EpsC* complementation control. Two-way ANOVA was used for Fig. 7. Significance is indicated as *P ≤ 0.05, **P ≤ 0.01, ***P ≤ 0.001, ****P ≤ 0.0001.

## SUPPLEMENTAL MATERIAL

Supplemental materials jb.00144-25-s0001.pdf Supplemental method, tables, and figures.

ASM does not own the copyrights to Supplemental Material that may be linked to, or accessed through, an article. The authors have granted ASM a non-exclusive, world-wide license to publish the Supplemental Material files. Please contact the corresponding author directly for reuse.
