# Supplementary material for: The PDZ domain of EpsC is required for extracellular secretion of VesB by the Type II secretion system in Vibrio cholerae
Source: J Bacteriol. 2025 Jul 14;207(8):e00144-25. doi: 10.1128/jb.00144-25 (PMC12369372; doi:10.1128/jb.00144-25)
Supplement: Supplemental materials — Supplemental method, tables, and figures. [file jb.00144-25-s0001.pdf]

The PDZ domain of EpsC is required for extracellular secretion of VesB by the Type II Secretion System in *Vibrio cholerae*

Austin Shannon[a], Tanya Johnson[a*], Cameron S. Roberts[a], Catherine T. Chaton[b], Konstantin V. Korotkov[b], Maria Sandkvist[a#]

SUPPLEMENTAL METHODS

Cholera toxin expression and Western blot

AKI induction was performed as previously described (1) but with modifications. Briefly, 10mL of AKI media supplemented with fresh $NaHCO_3$ was inoculated with *Vibrio cholerae* and left stationary at 37°C for 4 h before reducing the volume to 3mL and shaking the cultures at 37°C overnight to induce Cholera toxin expression. Culture supernatants were then isolated via centrifugation and proteins were concentrated via protein precipitation with an equal volume of pyrogallol red-molybdate-methanol (PRMM) solution (2). The mixture was incubated for 2 h at ambient temperature followed by a 16 h incubation at 4°C as described previously (3). Precipitated proteins were pelleted at 10,000xg for 1 h at 4°C, washed with cold acetone and centrifuged twice before air-drying. Protein pellets were resuspended in 1x Laemmli sample buffer supplemented with DTT and heated for 20min at 90°C. OD-corrected samples were then loaded into a 4-12% Bis-Tris gradient gel along with 250ng of purified Cholera toxin (Sigma-Aldrich: C8052-.5mg) as a positive control. After transferring the gel to nitrocellulose, the subsequent Western blot was blocked with Tris-buffered saline plus 5% Skim milk and detected with a rabbit anti-Cholera toxin primary (Sigma) followed by a goat anti-rabbit HRP secondary. The Western blot was developed with Pierce™ ECL2 chemiluminescence substrate and detected on the Amersham Typhoon platform with Cy2 settings at 375 V.

Table S1. Quantitative LC-MS/MS reveals proteins significantly changed in *epsCΔPDZ* mutant supernatants

Protein	MW	N16961✦			N16961 *epsCΔPDZ*✦			Fold Change (WT/ΔPDZ)	p-value
		1	2	3	1	2	3		
VesB	43 kDa	0.00118	0.000946	0.00118	6.23E-05	3.55E-05	2.14E-05	28	0.00018
VC_A0212	40 kDa	0.00104	0.00115	0.000862	0.000484	0.000223	0.000262	3.2	0.004
LuxP	41 kDa	0.00135	0.00134	0.000962	0.000602	0.000293	0.000537	2.6	0.0095
VC_2622	29 kDa	0.00223	0.00199	0.00186	0.0012	0.000762	0.000581	2.4	0.0053
LptE	24 kDa	0.000837	0.000934	0.00101	0.000704	0.000663	0.000651	1.4	0.0079
RpsG	18 kDa	0	6.28E-05	0.000179	0.00148	0.00148	0.000868	0.06	0.0047
PspA	25 kDa	0	0	0	6.89E-05	0.000054	0.000103	ϴ	0.0064
VC_2013	52 kDa	0	0	0	0.000118	0.000183	0.000186	ϴ	0.0019

✦ Normalized Spectral Abundance Factor (NSAF) for each pooled sample (pool ‘1’, ‘2’, or ‘3’)

ϴ Undefined fold-change with 0 in the numerator

Table S2. Primers used in this study

Primer name	Genotype / Purpose	Forward	Reverse
AmpR	β-lactamase into pMMB (EcoRI and HindIII)	CGCAGAGAATTCGATATACATACTATAACAAGCATGC	CGCAGAAAGCTTGAGAATAGAAGAGATAGAGAAG
AmpR-VesB IG	β-lac:B chimera	CTCACTGATTAAGCATTGGCACACTAACCAGCTTAGCTATGATC	GCTAAGCTGGTTAGTGTGCCAATGCTTAATCAGTGAGGC
EcoRI + epsC RBS Fwd		taaGcagaatTctcTGCGCAATATGAAGCGTAAAAAGTACAG	
eps upstream 5' Fwd		GTACAAACAGATTTGCGC	
EpsC DS Rev			GCCTCACTAAGGCGTCGTTAC
epsC N217 + Ec V171	GspCEc chimera	GCAATCGCACGAAATGTACTCGCTGATTATA	AtcagCgaGtacATTTCGTGCGATTGCTTC
epsC N217 + GspCAb R195	GspCAb chimera	gcAcGaAAtCGAGAGCAGTATCTCCG	AtacTgCTctcGATTTCGTGCGATTGCTTC
epsC N217 + PulC P190	PulC chimera	aTCgcacgAaAtCCGCAAAATATCCTCAC	gATaTttTGCGGATTTCGTGCGATTGCTTC
EpsC PDZ delta 2 Rev + BamHI			TaAGCaggAtccTTaAATATATACATCATGTTGTTG
EpsC PDZ delta 3 Rev + BamHI			taagcAggATccttaATATACATCATGTTGTTGACC
EpsC PDZ delta 4 Rev + BamHI			TaAgcAggatcctTATACATCATGTTGTTGACCATC
PulC 3' + BamHI Rev	PulC chimera		gaggatcctGCttTTATTCATCCCCCAGC
GspCAb 3' + BamHI Rev	GspCAb chimera		aagCagGaTcctcCTAAAAATTTTGTTGTATGGTC
GspCEc 3' +BamHI Rev	GspCEc chimera		GagGatccTGcTTTTATTGAGTCCTTTCATG
PDZ Deletion Rev			CGCGACGAATAATTTCCGC
pmmB Far04 Rev			CTCATGAGCGGATACATATTTGAATG
pmmB (03 fwd or 04 rev)	MCS amplification	TGGCTGTGCAGGTCGTAAATCAC	TACTCAGGAGAGCGTTCACCGACAAACAAC
Seq EpsC 5' end-Rev			TTTGTTCAGCCGAGACGA
VesB Delta20 Rev + BamHI			TaagcAGGATCCTCAACCGCCAGAAGAGGCAC
VesA S221A		CTGGGGATgCCGGAGGTCCTGTCTATT	GACCTCCGGcATCCCCAGAGCAGGTG
VesB GG6His		CATCATCACCATCATCACTGATTACCTATCCGAGATCTG	GTGATGATGGTGATGATGACCGCCAGAAGAGGCACTTG
VesC GG6His		cAtcAtcAcTGATCTATCGATAGCCAGCATGC	gTgaTgaTgACCGCCGCCACTATC
VesB L270 to D371	Ig removal from VesB	acacTAacCAgCTtGATACTTCGCCTTTTGCC	aagGcgaAgtatcAAGCTGGTTAGTGTGCTTTG
VesC CTERM + BamHI Rev			GaggATCctgcttTCAGACGCGTTGACGAC
VesC G526 STOP + BamHI Rev			GaggATCctgcttTCAACCGCCGCCACTATC
VesA GG6His		PHOS / catcaccattgaactttatgatcaaggtgtg	PHOS / gtgatggtgtcctccagatgagctctc
AhPDZfus	ExeC chimera	ATCGGTGATCTTGCCCGGATTTCGTGCGATTGCTTC	ATCGCACGAAATCCGGGCAAGATCACCGATTACCTC
CxlongTop	XcpP CC chiimera	ATCGCACGAAATCCGTTCCCGCACCGTTCCGGGGGA	
CxlongBot	XcpP CC chiimera		GGAACGGTGCGGGAACGGATTTCGTGCGATTGCTTC
XcpPend	XcpP CC chiimera		CGCGTCGACTCAGTCGCTTTCCGTAGGCGTGG
EpsC304stop			CGCCTGCAGTTATTGAATATATACATCATGTTG
EpsC300stop			CGCCTGCAGTTAATCATGTTGTTGACCATCACG
EpsCPDZtop	HR deletion A182 FWD	GATGCTCCGAAAGACTACACCGCGCCTGCG	
EpsCPDZbot	HR deletion K107 REV		CGCGGTGTAGTCTTTCGGAGCATCCACCAC
EpsCPDZN top	Removal of 203-218 A1 helix	GCTGTGCCGCAGGAAATTTTTCAATATGTG	
EpsCPDZN bot	Removal of 203-218 A1 helix		TTGAAAAATTTCCTGCGGCACAGCATTAGG
EpsC 177 stop	PDZ deletion		CGCCTGCAGTTAGTCTAACCCTTCAAGCATCAAG
BLACIGVESA	β-lac:A chimera	tcactgattaagcattggAATGGATTAGAGACGCCCAAG	ggcgtctctaatccattCCAATGCTTAATCAGTGAGGC
BLACIGVESC	β-lac:C chimera	tcactgattaagcattggCTAGGTCTATCCTATCGCCAAAAAC	GGCGATAGGATAGACCTAGCCAATGCTTAATCAGTGAGGC
birA_Nco		GTTCCATGGCTAAGGATAACACCGTGCCACTG	
birA_Hind			CTCAAGCTTATTTTTCTGCACTACGCAGG
PDZ_bio_Nco		ggttccatgGGTCTGAACGATATCTTCGAAGCTCAGAAAATCGAATGGCACGAA	
	Avi-tagged PDZ	AATGCTGTGCCGCAGTTTGAGGATAAAGTGGATGCAATTC	
PDZ_Hind	Avi-tagged PDZ		CCGCAAGCTTAAAATTGAATATATAC

Table S3. Strains and plasmids used in this study

#	Strain Background	Resistance	Plasmid	Notes
**1**	MC1061			For cloning and EpsC/GspCEc chimera
**2**	MM294	Kan	pRK2013	For conjugation
**3**	3083	Amp	pMMB67EH	
**4**	3083 *epsCΔPDZ*	Amp	pMMB67EH	
**5**	3083 *epsC::kan* ("*ΔepsC*")	Amp, Kan	pMMB67EH	
**6**	3083 *epsC::kan* ("*ΔepsC*")	Amp, Kan	pMMB67EH + *epsC*	
**7**	3083 *epsC::kan* ("*ΔepsC*")	Amp, Kan	pMMB67EH + *epsCΔTM*	
**8**	3083 *epsC::kan* ("*ΔepsC*")	Amp, Kan	pMMB67EH + *epsCΔHR*	
**9**	3083 *epsC::kan* ("*ΔepsC*")	Amp, Kan	pMMB67EH + *epsCΔPDZ*	This study
**10**	3083 *epsC::kan* ("*ΔepsC*")	Amp, Kan	pMMB67EH + *epsCΔ203-219*	This study
**11**	3083 *epsC::kan* ("*ΔepsC*")	Amp, Kan	pMMB67EH + *epsCΔ219-223*	This study
**12**	3083 *epsC::kan* ("*ΔepsC*")	Amp, Kan	pMMB67EH + *epsC(1-304)*	This study
**13**	3083 *epsC::kan* ("*ΔepsC*")	Amp, Kan	pMMB67EH + *epsC(1-303)*	This study
**14**	3083 *epsC::kan* ("*ΔepsC*")	Amp, Kan	pMMB67EH + *epsC(1-302)*	This study
**15**	3083 *epsC::kan* ("*ΔepsC*")	Amp, Kan	pMMB67EH + *epsC(1-301)*	This study
**16**	3083 *epsC::kan* ("*ΔepsC*")	Amp, Kan	pMMB67EH + *epsC(1-300)*	This study
**17**	3083 *epsC::kan* ("*ΔepsC*")	Amp, Kan	pmmB67EH + *epsC/ExeC PDZ*	This study
**18**	3083 *epsC::kan* ("*ΔepsC*")	Amp, Kan	pmmB67EH + *epsC/PulC PDZ*	This study
**19**	3083 *epsC::kan* ("*ΔepsC*")	Amp, Kan	pmmB67EH + *epsC/GspCEc PDZ*	This study
**20**	3083 *epsC::kan* ("*ΔepsC*")	Amp, Kan	pmmB67EH + *epsC/GspCAb PDZ*	This study
**21**	3083 *epsC::kan* ("*ΔepsC*")	Amp, Kan	pmmB67EH + *epsC/XcpP CC*	This study
**22**	N16961			
**23**	N16961 *epsCΔPDZ*			This study
**24**	N16961	Amp	pMMB67EH	
**25**	N16961 *vesA::cm*, *vesB::kan*, *ΔvesC*, ("NΔvesABC")	Amp, Kan, Cm	pMMB67EH	
**26**	N16961 *vesA::cm*, *vesB::kan*, *ΔvesC*, ("NΔvesABC")	Amp, Kan, Cm	pMMB67EH + *vesB*	
**27**	N16961 *vesA::cm*, *vesB::kan*, *ΔvesC*, ("NΔvesABC") *epsCΔPDZ*	Amp, Kan, Cm	pMMB67EH + *vesB*	This study
**28**	3083 *vesB::kan ("ΔvesB")*	Amp, Kan	pMMB67EH	
**29**	3083 *vesB::kan ("ΔvesB")*	Amp, Kan	pMMB67EH + *vesB*	
**30**	3083 *vesB::kan ("ΔvesB")*	Amp, Kan	pMMB67EH + *vesBΔ20*	
**31**	3083 *vesB::kan ("ΔvesB")*	Amp, Kan	pMMB67EH + *vesB(S221A)*	
**32**	3083 *vesB::kan ("ΔvesB")*	Amp, Kan	pMMB67EH + *vesB(S221A)*Δ20	
**33**	3083 *vesB::kan ("ΔvesB")*, *epsCΔPDZ*	Amp, Kan	pMMB67EH	This study
**34**	3083 *vesB::kan ("ΔvesB")*, *epsCΔPDZ*	Amp, Kan	pMMB67EH + *vesB*	This study
**35**	3083 *vesB::kan ("ΔvesB")*, *epsCΔPDZ*	Amp, Kan	pMMB67EH + *vesBΔ20*	This study
**36**	3083 *vesB::kan ("ΔvesB")*, *epsCΔPDZ*	Amp, Kan	pMMB67EH + *vesB(S221A)*	This study
**37**	3083 *vesB::kan ("ΔvesB")*, *epsCΔPDZ*	Amp, Kan	pMMB67EH + *vesB(S221A)*Δ20	This study
**38**	3083 *epsCΔPDZ*	Amp	pMMB67EH + *vesB*	This study
**39**	3083	Amp	pMMB67EH + *vesA(S221A)*6His	This study
**40**	3083	Amp	pMMB67EH + *vesB(S221A)*6His	This study
**41**	3083	Amp	pMMB67EH + *vesC(S225A)*6His	This study
**42**	3083 *epsCΔPDZ*	Amp	pMMB67EH + *vesA(S221A)*6His	This study
**43**	3083 *epsCΔPDZ*	Amp	pMMB67EH + *vesB(S221A)*6His	This study
**44**	3083 *epsCΔPDZ*	Amp	pMMB67EH + *vesC(S225A)*6His	This study
**45**	3083 *epsC::kan* ("*ΔepsC*")	Amp, Kan	pMMB67EH + *vesA(S221A)*6His	This study
**46**	3083 *epsC::kan* ("*ΔepsC*")	Amp, Kan	pMMB67EH + *vesB(S221A)*6His	This study
**47**	3083 *epsC::kan* ("*ΔepsC*")	Amp, Kan	pMMB67EH + *vesC(S225A)*6His	This study
**48**	N16961	Cm	pBBR + rpoEP2::lux	Plasmid from Sikora et al, 2007
**49**	N16961 *epsCΔPDZ*	Cm	pBBR + rpoEP2::lux	Plasmid from Sikora et al, 2007
**50**	N16961 *vesB::kan, epsCΔPDZ*	Cm, Kan	pBBR + rpoEP2::lux	Plasmid from Sikora et al, 2007
**51**	N16961 *ΔvesC*, *epsCΔPDZ*	Cm	pBBR + rpoEP2::lux	Plasmid from Sikora et al, 2007
**52**	N16961 *vesB::kan*, *ΔvesC, epsCΔPDZ*	Cm, Kan	pBBR + rpoEP2::lux	Plasmid from Sikora et al, 2007
**53**	N16961 Δ*epsD*	Cm	pBBR + rpoEP2::lux	Plasmid from Sikora et al, 2007
**54**	N16961	Kan	pMMB67EH(kan res)	This study
**55**	N16961	Kan, Amp	pMMB67EH(kan res) + β-lac	This study
**56**	N16961	Kan, Amp	pMMB67EH(kan res) + β-lac+Ig(B)	This study
**57**	N16961 *epsCΔPDZ*	Kan	pMMB67EH(kan res)	This study
**58**	N16961 *epsCΔPDZ*	Kan, Amp	pMMB67EH(kan res) + β-lac	This study
**59**	N16961 *epsCΔPDZ*	Kan, Amp	pMMB67EH(kan res) + β-lac+Ig(B)	This study
**60**	N16961 *vesA::cm*	Cm		
**61**	N16961 *vesB::kan*	Kan		
**62**	N16961 *ΔvesC*			
**63**	N16961 *vesA::cm*, *vesB::kan*	Cm, Kan		
**64**	N16961 *ΔHAP*			
**65**	N16961 *ΔvesC Δeps*			
**66**	3083	Amp	pMMB67EH + *vesBΔIg*-6his	This study
**67**	3083 *epsCΔPDZ*	Amp	pMMB67EH + *vesBΔIg*-6his	This study
**68**	NovaBlue	Kan	pRSF-NT	For cloning; from Korotkov et al. 2013 (PMID23820381)
**69**	T7 express::pRARE2	Kan, Cm	pRSF-bioPDZ	Expression of PDZ domain with AviTag
**70**	T7 express::pRARE2	Kan, Cm	pKV1622	For expression of BirA biotin ligase
**71**	Klebsiella pneumoniae KPPR1			EpsC/PulC chimera
**72**	Acinetobacter baumannii 17971			EpsC/GspCAb chimera
**73**	Pseudomonas aeruginosa PA01			EpsC/XcpP chimera
**74**	XL1	Carb	pPH14.5 exeCD in pBS	EpsC/ExeC chimera

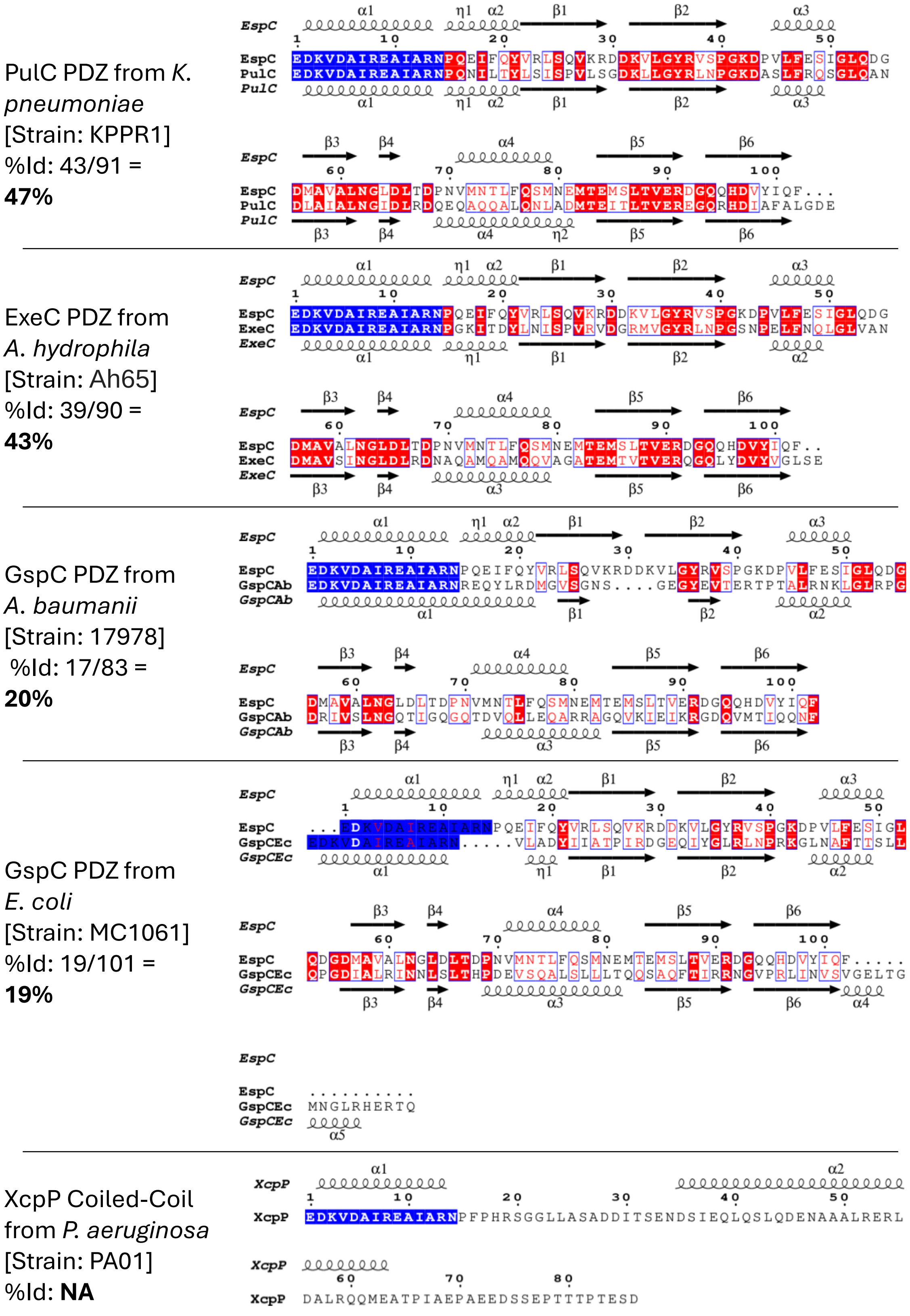

Figure S1. Comparison of PDZ domains used in EpsC chimera analysis. Pairwise structure-based alignments of PDZ of EpsC chimera. Percent identity is noted as the portion of exact matches (red) in the total number of amino acids from the non-*V. cholerae* homologue. Blue indicates the C-terminal region of EpsC included in each of the chimeric constructs with residue "1" in each depiction representing E204 of EpsC. N-terminal EpsC residues 1-203 are also present in each chimera but are excluded here for brevity. Secondary structures are indicated. The structure-based sequence alignments were performed using the Dali server (4) and rendered using ESPript 3.0 (5).

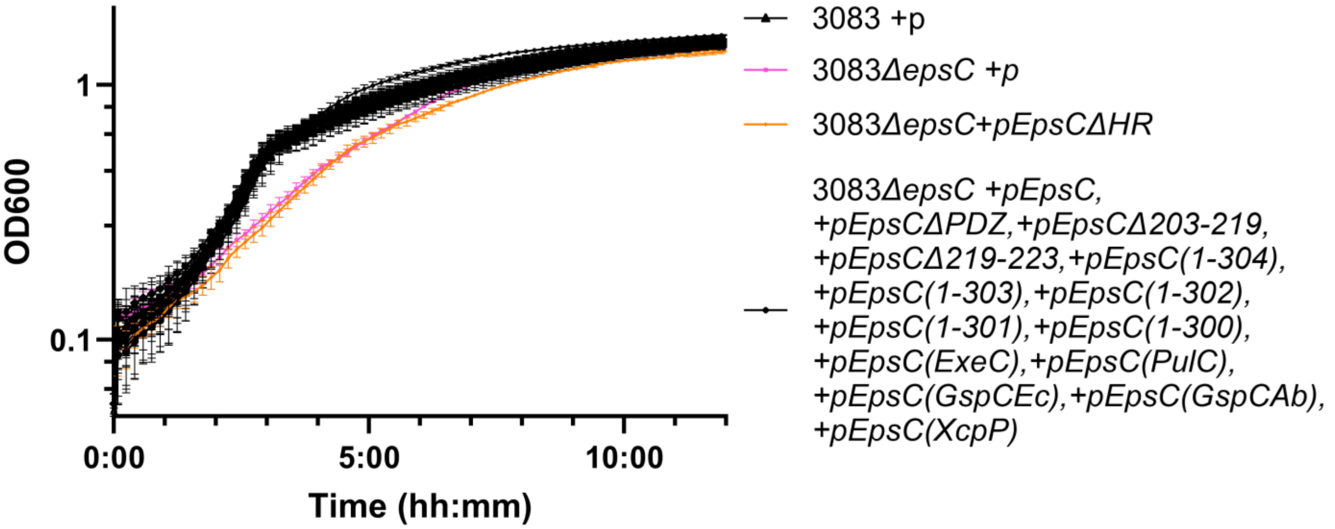

Figure S2. EpsC with chimeric or truncated PDZ complements *V. cholerae* growth. OD600 measurements of *V. cholerae* strains in LB and carbenicillin at 37C over time. Data represent the mean +/- the standard deviation of two biological replicates measured in technical triplicate (n=2).

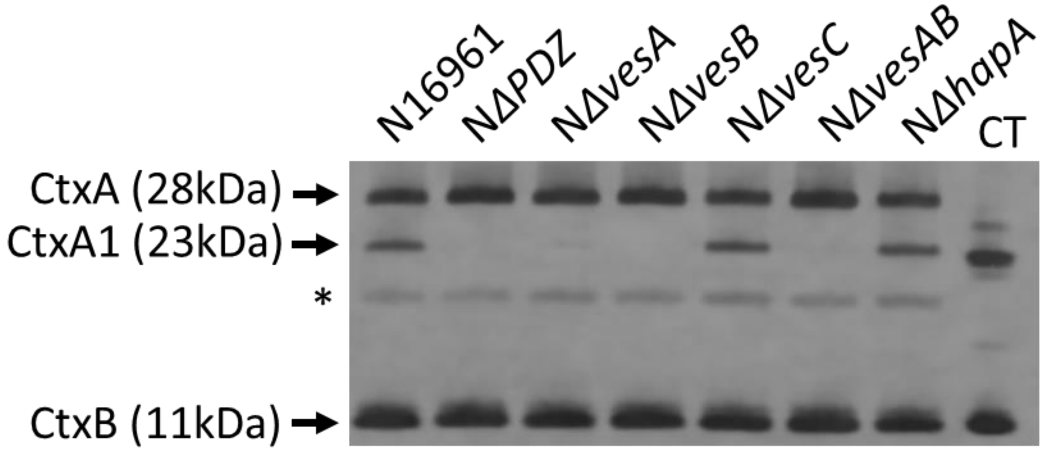

Figure S3. CT secretion is not PDZ-dependent, but CtxA cleavage is. Cholera toxin was detected in concentrated culture supernatants of different *V. cholerae* strains after PRMM precipitation (1) and sample reduction with DTT. A representative Western blot is shown from three separate experiments (n=3). "CT" indicates 250ng of purified cholera toxin used as a positive control. Sizes of the CtxA subunit, CtxB subunit, and the proteolytically cleaved CtxA subunit, CtxA1, are each indicated. A cross-reactive band is also indicated with an asterisk.

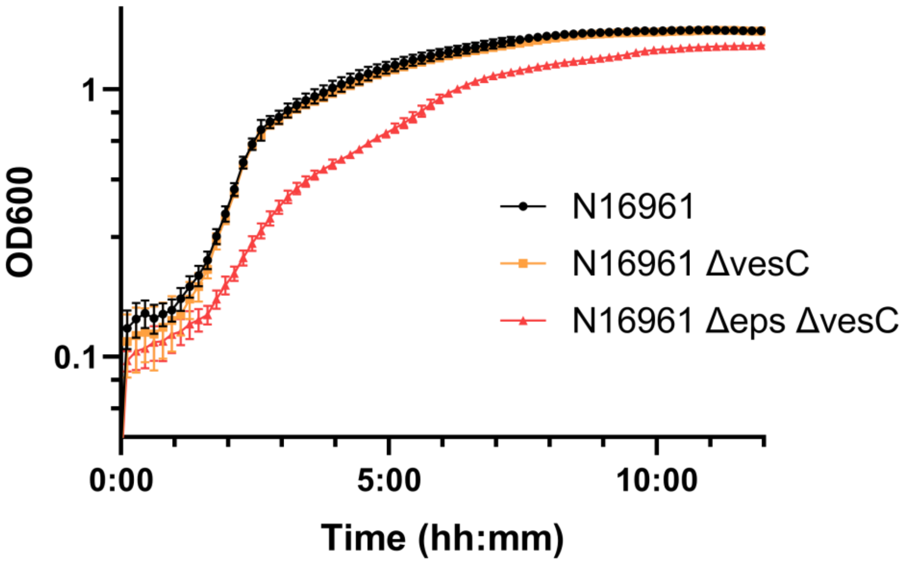

Figure S4. VesC is not solely responsible for the poor *Δeps* growth phenotype in *V. cholerae*. OD600 measurements of *V. cholerae* strains in LB at 37C over time. Data represent the mean +/- the standard deviation of two biological replicates measured in technical triplicate.

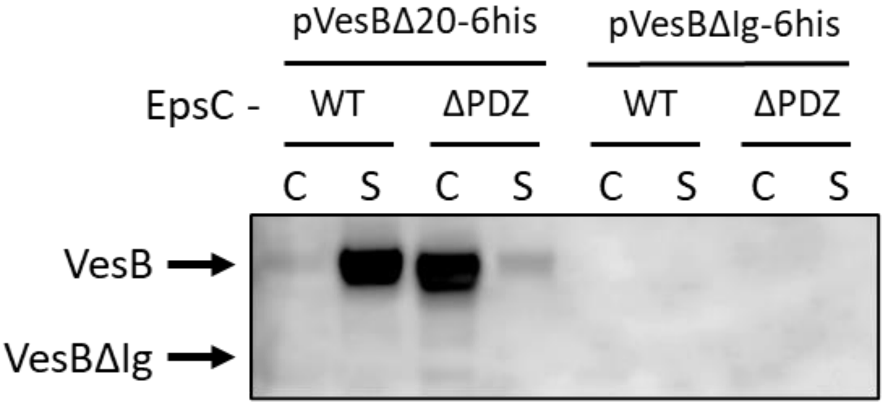

Figure S5. VesBΔIg is unstable. Western blot of *V. cholerae* cell (C) and culture supernatant (S) fractions during log-phase growth induced with 15uM IPTG in LB + carbenicillin and detected with anti-6his antibody. This blot is representative of two separate experiments (n=2).

References

1. Iwanaga M, Yamamoto K, Higa N, Ichinose Y, Nakasone N, Tanabe M. 1986. Culture Conditions for Stimulating Cholera Toxin Production by Vibrio cholerae O1 El Tor. Microbiol Immunol 30:1075–1083.
2. Caldwell RB, Lattemann CT. 2004. Simple and Reliable Method To Precipitate Proteins from Bacterial Culture Supernatant. Appl Environ Microbiol 610–612.
3. Sikora AE, Zielke RA, Lawrence DA, Andrews PC, Sandkvist M. 2011. Proteomic Analysis of the Vibrio cholerae Type II Secretome Reveals New Proteins, Including Three Related Serine Proteases. J Biol Chem 286:16555–16566.
4. Holm L. 2022. Dali server: structural unification of protein families. Nucleic Acids Research 50:W210–W215.
5. Robert X, Gouet P. 2014. Deciphering key features in protein structures with the new ENDscript server. Nucleic Acids Research 42:W320–W324.
